# Effect of population-approach programs promoting salt reduction and potassium intake in Japan: the Population-based Sodium/Potassium Improvement Program (PoSPIP)

**DOI:** 10.1038/s41440-026-02662-0

**Published:** 2026-05-14

**Authors:** Takashi Hisamatsu, Minako Kinuta, Takayoshi Ohkubo, Takuya Tsuchihashi, Katsushi Yoshita, Yukari Takemi, Hitomi Hayabuchi, Yukiko Okami, Kaori Kitaoka, Keiko Sakaguchi, Atsushi Hozawa, Tomonori Okamura, Hiroshi Itoh, Hiromi Rakugi, Koichi Node, Katsuyuki Miura

**Affiliations:** 1 Department of Public Health, Okayama University Graduate School of Medicine, Dentistry and Pharmaceutical Sciences, Okayama, Japan; 2 Department of Food Sciences and Nutrition, Mukogawa Women’s University, Nishinomiya, Japan; 3 Department of Hygiene and Public Health, Teikyo University School of Medicine, Tokyo, Japan; 4 Cardiovascular Center, Steel Memorial Yawata Hospital, Kitakyushu, Japan; 5 Department of Public Health, Nutrition, School of Human Life and Ecology, Osaka Metropolitan University, Osaka, Japan; 6 Faculty of Nutrition, Kagawa Nutrition University, Sakado, Japan; 7 Graduate School of Health and Environmental Sciences, Fukuoka Women’s University, Fukuoka, Japan; 8 Gunma University Center for Food Science and Wellness, Maebashi, Japan; 9 NCD Epidemiology Research Center, Shiga University of Medical Science, Otsu, Japan; 10 Section of Research Collaboration and Partnership, Center for Private-Public-Academic Collaboration Research, National Institutes of Biomedical Innovation, Health and Nutrition, Settsu, Japan; 11 Faculty of Nursing and Nutrition, Shukutoku University, Chiba, Japan; 12 Division of Epidemiology, School of Public Health, Tohoku University Graduate School of Medicine, Sendai, Japan; 13 Department of Preventive Medicine and Public Health, Keio University School of Medicine, Tokyo, Japan; 14 Japanese Society of Hypertension, Tokyo, Japan; 15 The Center for Preventive Medicine, Keio University, Tokyo, Japan; 16 Osaka Rosai Hospital, Sakai, Japan; 17 Department of Cardiovascular Medicine, Saga University, Saga, Japan

**Keywords:** Implemental hypertension, Sodium, Potassium, Urinalysis, Food environment

## Abstract

Reducing sodium intake in populations is essential, but insufficient for preventing and managing high blood pressure, while the importance of increasing potassium intake is overlooked. We investigated the effects of 1-year population-approach programs (2021–2022) promoting salt reduction and potassium intake using urinalysis feedback and food environment improvement. This retrospective observational study included 7649 participants (mean age, 54.0 years; 45.3% women) from 11 municipalities and 4 workplaces. Outcomes in intensive support programs—including urinary sodium, potassium, and sodium-to-potassium (Na/K) ratio measurements with feedback, dietary promotion, and food environment improvement—were compared with standard support programs providing usual health guidance. In linear regression adjusted for demographics, lifestyle factors, and medical history, the reduction in urinary Na/K ratio was greater in the intensive support group (*n* = 4064) than in the standard support group (*n* = 3585) (mean difference −0.14 [95% confidence interval, −0.27 to −0.01]). Although estimated potassium intake decreased in both groups, the decline was smaller in the intensive support group (mean difference 31 [12 to 51] mg/day). Estimated salt intake did not differ between the groups. The intensive support group showed greater increases in diastolic blood pressure and high-density lipoprotein cholesterol and smaller increases in blood glucose, as well as greater reductions in hemoglobin A1c and Salt Check Sheet scores. Mean differences between the groups for endpoints were not heterogeneous across intensive support program types. Our findings support the development of hypertension prevention and management strategies that promote healthier dietary behaviors and can be implemented in community and workplace settings, with broad public health applicability.

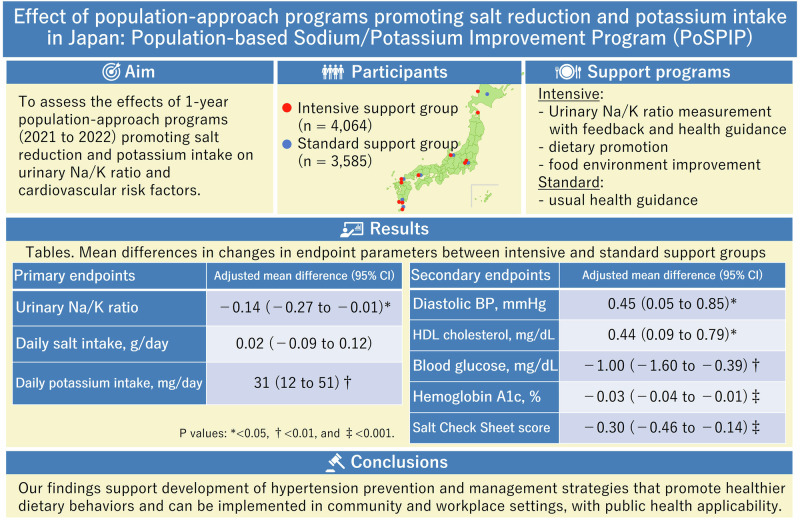

## Introduction

Hypertension is the leading risk factor for premature mortality from cardiovascular diseases (CVDs) and is ranked first as a cause of disability-adjusted life years. In Japan, the prevalence of hypertension remains high, with an estimated 43 million hypertensive individuals, and most (31 million, 72%) of them are poorly controlled [1]. Several factors contribute to the development of hypertension, among which sodium (Na) and potassium (K) intake are key modifiable determinants [2].

The growing body of evidence highlights a positive association between blood pressure (BP) and Na intake, as well as an inverse relationship with K intake, based on findings from observational studies [3] and randomized clinical trials focused on dietary interventions [4]. Additionally, the urinary sodium-to-potassium (Na/K) ratio—determined by dividing the Na concentration by the K concentration (both measured in mmol/L) from 24-h or casual urine samples as an indicator of the relative balance between Na and K intakes [5]—has demonstrated a stronger positive association with elevated BP than urinary Na or K excretion alone [3, 6]. However, in the nationwide health check-up and guidance programs in municipalities and workplaces across Japan, for the prevention and management of high BP, population-based strategies have primarily focused on Na reduction, and the importance of increasing K intake is often overlooked [7]. Recently, the objective assessment of Na and K intakes using urine samples is expected to effectively promote Na reduction and an increased K intake [5]. In addition, food-environment interventions aimed at reducing excessive salt intake seek to create health-promoting settings through food reformulation combined with consumer education and have demonstrated effectiveness at the population level through industry–academia–government collaboration [8].

The Japan Society of Hypertension conducted the Population-based Sodium/Potassium Improvement Program (PoSPIP) in multiple municipalities and workplaces from 2020 to 2022, as part of the Verification Programs for Prevention and Health Promotion by the Ministry of Health, Labour and Welfare of Japan. The PoSPIP focused on urinary Na and K measurement and improving the food environment to promote healthier dietary behaviors [9]. Utilizing the data from this project, we investigated the effects of 1-year comprehensive intensive support programs between 2021 and 2022 aimed at promoting salt (Na) reduction and increased K intake across populations on changes in the urinary Na/K ratio, daily salt and K intakes estimated by casual urine samples, and other cardiovascular risk factors. These findings provide practical insights for the development of population-based hypertension prevention and management strategies.

Point of view

- Clinical relevance:
  The programs were associated with a reduction in the urinary Na/K ratio and a positive between-group difference in K intake, along with improvements in several CVD risk factors. While the effect size was modest, these findings support the development of population-approach strategies to improve diet-related risk profiles relevant to hypertension prevention, which can be implemented in community and workplace settings with broad applicability in public health promotion.
- Future direction:
  Further studies are warranted to evaluate whether sustained implementation of such population-approach programs leads to improvements in population BP distribution and clinical outcomes, particularly in community and workplace settings.
- Consideration for the Asian population:
  Given the persistently high Na and insufficient K intake in many Asian countries, population-based approaches promoting Na reduction and K improvement are essential for effective BP control.

## Materials and methods

### Study design and participants

Our study was a 1-year retrospective observational study based on data collected from individuals who participated in population-approach programs conducted by the Japan Society of Hypertension [9]. Eleven municipal health insurers—Mashike Town and Higashikagura Town in Hokkaido Prefecture; Higashidori Village in Aomori Prefecture; Kure City in Hiroshima Prefecture; Kitakyushu City and Umi Town in Fukuoka Prefecture; and Makurazaki City, Tarumizu City, Kinko Town, Nishino-omote City, and Nakatane Town in Kagoshima Prefecture—along with four workplace participants—YKK Corporation, Fujifilm Wako Pure Chemical Corporation, Toyo Aerosol Industry Co., Ltd., and Sanofi K.K. Kawagoe Factory (currently Kawagoe Seiyaku Co., Ltd.)—expressed their willingness to participate as program fields. Before the programs began, the program committee categorized the participating municipalities and workplaces into two groups (intensive support group and standard support group) to ensure that both groups had similar characteristics [9]. The intensive support group included Mashike Town, Higashidori Village, Kure City (East Health Center attendees), Yahatahigashi and Yahatanishi Wards of Kitakyushu City, Umi Town, Makurazaki City, Kinko Town, Nakatane Town, YKK Corporation’s Kurobe Plant, Fujifilm Wako Pure Chemical Corporation, and Toyo Aerosol Industry Co., Ltd. The standard support group included Higashikagura Town, Kure City (West Health Center attendees), Kokurakita and Moji Wards of Kitakyushu City, Tarumizu City, Nishino-omote City, other plants of the YKK Corporation in Kurobe, and the Sanofi K.K. Kawagoe Factory.

**Fig. 1:**
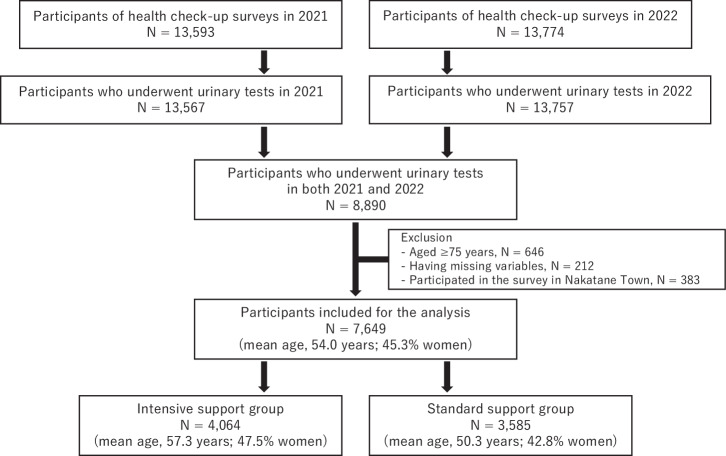
Flowchart of the study participants

The flowchart of study participants is shown in Fig. 1. In brief, a total of 13,567 and 13,757 participants underwent the health check-up surveys, including the urinary tests in 2021 and 2022, respectively. Among them, 8890 participants underwent the surveys in both 2021 and 2022. We excluded 646 participants aged 75 years or older because we limited the analysis to individuals eligible for the health check-up and health guidance program, which targets those under 75 years of age; 212 participants with missing variables; and 383 participants from Nakatane Town because of differences in the urine collection methods between years. Consequently, we included 7649 participants (mean age, 54.0 years; 45.3% women), comprising 4064 in the intensive support group and 3585 in the standard support group, in the present analysis. Our study was approved by the institutional review board committee of the Japanese Society of Hypertension (approval number: 2023-001) and Okayama University Graduate School of Medicine, Dentistry and Pharmaceutical Sciences and Okayama University Hospital (approval number: 2309-030). The institutional review board waived the informed consent requirement for participants because of the use of deidentified data and the secondary use of existing data.

### Intensive and standard support programs

For all participants in both the intensive and standard support groups, urinary Na and K measurements were performed using casual urine samples on the day of their health check-up, and feedback was provided at a later date. Additionally, the intensive support programs for participants undergoing health check-ups and guidance were implemented using the following methods [9]: (1) measuring the urinary Na/K ratio using casual urine samples (HEU-001F; OMRON Healthcare Co., Ltd., Kyoto, Japan) [10] and providing feedback immediately on-site along with health guidance using educational materials (i.e., textbooks, awareness posters, and videos) focusing on salt reduction and increasing K intake on the day of the health check-up; (2) utilizing textbooks or smartphone-based messaging apps (LINE: LY Corporation, Tokyo, Japan) equipped with features for continuous self-monitoring, information dissemination, goal setting, and evaluation of dietary habits, including salt reduction and increasing K intake; (3) offering repeated personalized health guidance on later days using the educational materials; and (4) conducting repeated measurements of the urinary Na/K ratio from casual urine samples on later days using a self-monitoring device (HEU-001F) [10] or a mail-in urine measurement service (Salt-Reduction Test ShioCheck +; Healthcare Systems Co., Ltd., Tokyo, Japan) [11]. Because of the circumstances of participating municipalities and workplaces, some intensive support programs were not implemented in certain municipalities or workplaces. Additionally, the intensive support group was also subjected to the food environment improvement programs. In contrast, the standard support program included usual health guidance alone.

The approach to improving the food environment focused on two key aspects: food availability and information availability [12]. These aspects are interrelated and mutually reinforcing. Accordingly, efforts to improve the food environment were categorized into three areas: (1) improving food availability and accessibility, (2) improving information availability and accessibility, and (3) creating momentum [13]. Specific initiatives were developed and implemented in each study field in the intensive support group. These initiatives were classified into two categories: required items, which were implemented across all study fields in the intensive support group, and voluntary items, which could be selected based on the circumstances of each study field in the intensive support group. Details of the initiatives are provided in Supplementary Table 1.

### Endpoint and covariate assessments

The endpoints were determined based on examinations conducted during the annual health check-ups and were predefined by the project committee as changes in the following parameters between the 2021 and 2022 surveys [9]. The primary endpoints were the urinary Na/K ratio (calculated by dividing the Na concentration by the K concentration, both measured in mmol/L) from casual urine samples and the estimated daily salt and K intakes based on the Tanaka formula [14] using casual urine samples. For urinary measurements in 2021 and 2022, early-morning urine samples collected at home were used for analyses from Mashike Town, Higashikagura Town, Kure City, Kitakyushu City, Umi Town, Tarumizu City, Kinko Town, Nishino-omote City, Fujifilm Wako Pure Chemical Corporation, Ltd., Toyo Aerosol Industry Co., Ltd., and Sanofi K.K. Kawagoe Factory, while daytime urine samples collected during health check-ups were used for analyses from Higashidori Village, Makurazaki City, and YKK Corporation. The secondary endpoints included systolic and diastolic BP, body weight, low-density lipoprotein (LDL) cholesterol, high-density lipoprotein (HDL) cholesterol, triglycerides, blood glucose, hemoglobin A1c, and the Salt Check Sheet score, derived from a questionnaire assessing salt intake, with lower scores indicating greater salt reduction [15].

A self-administered questionnaire was used to obtain information on demographics; lifestyle factors including smoking status, alcohol drinking, and physical activity; medication use; and medical history. BP was measured using a standard electric or automated sphygmomanometer on the right arm of seated participants after ≥5 min of rest. When multiple BP measurements were obtained, the lowest value was used for analysis because the number of BP measurements varied (e.g., once, twice, or three times) across study fields. Body mass index was calculated as weight (kg) divided by height squared (m^2^). Active exercise was defined as engaging in light to moderate exercise for more than 30 min at a time, at least twice per week, for over a year [16]. Hypertension was defined as systolic BP ≥ 140 mmHg, diastolic BP ≥ 90 mmHg, or antihypertensive medication use [17]. Dyslipidemia was defined as LDL cholesterol ≥140 mg/dL, triglycerides ≥150 mg/dL, HDL cholesterol <40 mg/dL, or lipid-lowering medication use [18]. Diabetes mellitus was defined as fasting blood glucose ≥126 mg/dL, hemoglobin A1c ≥ 6.5%, or antidiabetic medication use [19].

### Statistical analysis

The effect of the intensive support programs was calculated using the absolute mean difference in changes of each endpoint parameter from 2021 to 2022 between the intensive and standard support groups. Given that the baseline characteristics differed to some extent between the intensive and standard support groups, we applied the multivariable linear regression adjusted for age, sex, BMI, smoking status, alcohol drinking, exercise, hemoglobin A1c, medication for hypertension, diabetes, and dyslipidemia; history of stroke, heart disease, and chronic kidney disease; and study field type (municipality or workplace). For primary endpoints based on urinary indices, we further adjusted for the timing of the urinary test (morning at home or daytime during health check-up). We also assessed the differences in the effects across the types of intensive support programs. For the sensitivity analyses, stratified analyses were performed by sex, age group (18–39 years; 40–64 years; ≥65 years), quartile of baseline urinary Na/K ratio, estimated salt intake and estimated K intake, baseline alcohol drinking status, baseline BMI category ( <18.5; 18.5–24.9; ≥25.0 kg/m^2^), baseline status of hypertension and diabetes, and study field type (municipality or workplace). We additionally performed a sensitivity analysis stratified by the timing of urinary sampling (morning at home vs. daytime during health check-up) for the primary endpoints. We further tested for the heterogeneities in the associations between the intensive support programs and these variables in relation to the endpoints. All analyses were performed using Stata version 18 (StataCorp LLC, College Station, TX, USA). All statistical tests were two-tailed, with P values below 0.05 considered significant.

## Results

### Baseline characteristics and endpoint parameters

The baseline characteristics in the intensive and standard support groups are shown in Table 1. Participants in the intensive support group were older, had higher BMI levels, and had higher prevalences of women, active exercisers, and those with a history of stroke and heart disease than those in the standard support group. They were also more likely to use medication for hypertension, dyslipidemia, and diabetes mellitus; had a lower prevalence of current smokers; and were more likely to be from municipalities. The distributions of demographics and the primary and secondary endpoint parameters of participants in each study field are shown in Supplementary Table 2.

**Table 1:** Baseline characteristics in the intensive and standard support groups

	Intensive support *n* = 4064	Standard support *n* = 3585	*P* value
Age, years	57.3 (15.6)	50.3 (17.6)	<0.001
Women, *n* (%)	1930 (47.5)	1534 (42.8)	<0.001
Body mass index, kg/m^2^	23.1 (3.7)	22.8 (3.6)	0.002
Current smokers, *n* (%)	618 (15.2)	663 (18.5)	<0.001
Current drinkers, *n* (%)	2151 (52.9)	1823 (50.9)	0.070
Active exercise, *n* (%)	1454 (35.8)	1191 (33.2)	0.019
Medication use, *n* (%)			
Hypertension	1046 (25.7)	633 (17.7)	<0.001
Dyslipidemia	732 (18.0)	457 (12.8)	<0.001
Diabetes mellitus	255 (6.3)	163 (4.6)	0.001
Medical history, *n* (%)			
Stroke	112 (2.8)	67 (1.9)	0.010
Heart disease	212 (5.2)	124 (3.5)	<0.001
Chronic kidney disease	23 (0.6)	26 (0.7)	0.384
Participants from municipalities, *n* (%)	2903 (71.4)	1685 (47.0)	<0.001

Values are expressed as means (standard deviations) or numbers (percentages)

*P*-values were calculated using unpaired t-tests or chi-square tests

### Mean differences in changes in primary and secondary endpoints

The mean differences in changes from baseline to follow-up of each endpoint parameter between the intensive and standard support groups are shown in Table 2. The baseline means of the urinary Na/K ratio, estimated daily salt and K intakes, and systolic and diastolic BP were 3.71, 8.85 g/day, 1976 mg/day, 124.59 mmHg, and 75.05 mmHg, respectively, for the intensive support group and 3.63, 8.93 g/day, 2005 mg/day, 121.62 mmHg, and 74.05 mmHg, respectively, for the standard support group. For the primary endpoints, after adjustment for demographics, lifestyle factors, and medical history, the reduction in the urinary Na/K ratio was significantly greater in the intensive support group than in the standard support group (mean difference, −0.14 [95% confidence interval, −0.27 to −0.01]). Although estimated K intake decreased in both groups over time, the decline was significantly smaller in the intensive support group compared with the standard support group (mean difference, 31 [12 to 51] mg/day). There was no significant difference in the change in estimated salt intake between the two groups. For the secondary endpoints, the intensive support group showed significantly greater increases in diastolic BP (mean difference [95% confidence interval], 0.45 [0.05 to 0.85] mmHg) and HDL cholesterol (0.44 [0.09 to 0.79] mg/dL) and smaller increases in blood glucose (−1.00 [−1.60 to −0.39] mg/dL), as well as greater decreases in hemoglobin A1c (−0.03 [−0.04 to −0.01] %) and Salt Check Sheet scores (−0.30 [−0.46 to −0.14]), than those in the standard support group.

**Table 2:** Mean differences in changes in primary and secondary endpoint parameters between the intensive and standard support groups

	Means (95% CIs) of intensive support group, *n* = 4064			Means (95% CIs) of standard support group, *n* = 3585			Mean difference (95% CI)	Adjusted mean difference (95% CI)
	Baseline	Follow-up	Change	Baseline	Follow-up	Change		
Primary endpoints								
Urinary Na/K ratio	3.71 (3.64 to 3.79)	3.69 (3.61 to 3.76)	−0.03 (−0.11 to 0.06)	3.63 (3.55 to 3.71)	3.72 (3.64 to 3.80)	0.10 (0.01 to 0.18)	−0.12 (−0.24 to 0.00)*	−0.14 (−0.27 to −0.01)*
Estimated daily salt intake, g/day	8.85 (8.79 to 8.91)	8.79 (8.73 to 8.86)	−0.06 (−0.12 to 0.01)	8.93 (8.86 to 9.00)	8.86 (8.79 to 8.92)	−0.07 (−0.15 to 0.00)	0.02 (−0.08 to 0.12)	0.02 (−0.09 to 0.12)
Estimated daily K intake, mg/day	1976 (1962 to 1989)	1965 (1952 to 1979)	−10 (−23 to 3)	2005 (1990 to 2019)	1968 (1954 to 1983)	−36 (−50 to −23)	26 (8 to 45)†	31 (12 to 51)†
Secondary endpoints								
Systolic BP, mmHg	124.59 (124.07 to 125.11)	124.15 (123.64 to 124.66)	−0.44 (−0.84 to −0.05)	121.62 (121.07 to 122.18)	121.73 (121.19 to 122.27)	0.11 (−0.31 to 0.53)	−0.55 (−1.13 to 0.02)	−0.18 (−0.77 to 0.42)
Diastolic BP, mmHg	75.05 (74.71 to 75.40)	75.46 (75.12 to 75.80)	0.41 (0.14 to 0.68)	74.05 (73.68 to 74.41)	74.51 (74.14 to 74.87)	0.46 (0.17 to 0.75)	−0.05 (−0.45 to 0.34)	0.45 (0.05 to 0.85)*
Body weight, kg	60.70 (60.31 to 61.08)	60.57 (60.18 to 60.96)	−0.12 (−0.20 to −0.05)	61.43 (61.02 to 61.84)	61.51 (61.09 to 61.92)	0.08 (0.00 to 0.15)	−0.20 (−0.31 to −0.10)‡	−0.06 (−0.17 to 0.05)
LDL cholesterol, mg/dL	122.82 (121.86 to 123.77)	120.69 (119.77 to 121.62)	−2.12 (−2.73 to −1.51)	121.31 (120.29 to 122.32)	119.08 (118.10 to 120.07)	−2.23 (−2.88 to −1.58)	0.11 (−0.78 to 1.00)	0.73 (−0.18 to 1.65)
HDL cholesterol, mg/dL	64.75 (64.23 to 65.27)	65.48 (64.96 to 66.00)	0.73 (0.50 to 0.96)	64.83 (64.28 to 65.38)	64.66 (64.11 to 65.21)	−0.16 (−0.41 to 0.09)	0.89 (0.55 to 1.23)‡	0.44 (0.09 to 0.79)*
Triglycerides, mg/dL	108.18 (105.74 to 110.61)	105.40 (103.11 to 107.69)	−2.72 (−4.95 to −0.49)	106.04 (103.44 to 108.64)	104.33 (101.89 to 106.77)	−1.71 (−4.09 to 0.66)	−1.00 (−4.26 to 2.25)	−1.80 (−5.15 to 1.56)
Blood glucose, mg/dL	98.93 (98.33 to 99.54)	98.97 (98.39 to 99.54)	0.04 (−0.36 to 0.44)	95.88 (95.18 to 96.58)	96.57 (95.92 to 97.22)	0.91 (0.44 to 1.37)	−0.86 (−1.48 to −0.25)†	−1.00 (−1.60 to −0.39)†
Hemoglobin A1c, %	5.56 (5.54 to 5.58)	5.54 (5.52 to 5.56)	−0.02 (−0.03 to −0.01)	5.46 (5.44 to 5.48)	5.46 (5.44 to 5.48)	−0.01 (−0.01 to 0.00)	−0.01 (−0.03 to 0.00)*	−0.03 (−0.04 to −0.01)‡
Salt Check Sheet score	12.33 (12.20 to 12.47)	11.73 (11.59 to 11.87)	−0.60 (−0.71 to −0.50)	12.64 (12.50 to 12.79)	12.32 (12.18 to 12.46)	−0.31 (−0.42 to −0.20)	−0.30 (−0.45 to −0.14)‡	−0.30 (−0.46 to −0.14)‡

*BP* blood pressure, *CI* confidence interval, *HDL* high-density lipoprotein, *K* potassium, *LDL* low-density lipoprotein, *Na/K ratio* sodium-to-potassium ratio

Mean differences adjusted for age, sex, body mass index, smoking status, alcohol drinking, exercise, hemoglobin A1c, medication for hypertension, diabetes, and dyslipidemia, history of stroke, heart disease, and chronic kidney disease, and study field type. Further adjusted for the timing of the urinary test for the analysis of urinary indices

P values: *<0.05, †<0.01, and ‡<0.001

We also observed no statistically significant heterogeneities in the mean differences between the groups across the types of intensive support programs for the primary and secondary endpoints (all P values for heterogeneity >0.05) (Table 3). For example, between-group differences in the urinary Na/K ratio (−0.15 to −0.11) and between-group differences in the estimated K intake (30 to 38 mg/day) were consistently observed in the intensive support group compared with the standard support group across all types of intensive support programs. However, no significant differences in the change in estimated salt intake were observed between the groups across all types of intensive support programs.

**Table 3:** Mean differences in changes in primary and secondary endpoint parameters between the intensive and standard support groups across types of intensive support programs

Program type	On-site urinary Na/K ratio measurement with immediate feedback	Use of textbooks or apps for salt reduction and increased K intake	Repeated personalized health guidance	Repeated measurement of the urinary Na/K ratio		P for heterogeneity
				Self-monitoring device	Mail-in measurement service	
	*N* = 2912	*N* = 2708	*N* = 2936	*N* = 2826	*N* = 2931	
Primary endpoints						
Urinary Na/K ratio	−0.15 (−0.28 to −0.01)*	−0.11 (−0.24 to 0.03)	−0.13 (−0.27 to 0.00)	−0.13 (−0.27 to 0.01)	−0.15 (−0.28 to −0.01)*	0.933
Estimated daily salt intake, g/day	0.04 (−0.08 to 0.16)	0.08 (−0.04 to 0.20)	0.02 (−0.09 to 0.14)	0.02 (−0.10 to 0.14)	0.04 (−0.07 to 0.15)	0.574
Estimated daily K intake, mg/day	35 (14 to 57)†	34 (11 to 56)†	30 (9 to 52)†	34 (12 to 56)†	38 (18 to 59)‡	0.674
Secondary endpoints						
Systolic BP, mmHg	0.38 (−0.21 to 0.98)	0.38 (−0.23 to 0.98)	−0.12 (−0.74 to 0.49)	0.03 (−0.60 to 0.66)	−0.47 (−1.08 to 0.14)	0.055
Diastolic BP, mmHg	0.52 (0.10 to 0.94)*	0.51 (0.09 to 0.94)*	0.54 (0.10 to 0.97)*	0.63 (0.19 to 1.07)†	0.04 (−0.37 to 0.46)	0.583
Body weight, kg	−0.02 (−0.13 to 0.10)	−0.03 (−0.14 to 0.09)	−0.01 (−0.12 to 0.11)	0.02 (−0.10 to 0.14)	−0.05 (−0.16 to 0.07)	0.996
LDL cholesterol, mg/dL	−0.31 (−1.28 to 0.66)	−0.16 (−1.14 to 0.82)	0.85 (−0.12 to 1.82)	1.23 (0.24 to 2.21)*	−0.37 (−1.34 to 0.60)	0.119
HDL cholesterol, mg/dL	0.58 (0.22 to 0.94)†	0.66 (0.30 to 1.03)‡	0.62 (0.26 to 0.98)†	0.60 (0.23 to 0.97)†	0.10 (−0.27 to 0.47)	0.424
Triglycerides, mg/dL	−2.51 (−6.17 to 1.14)	−2.67 (−6.41 to 1.08)	−1.72 (−5.43 to 1.99)	−2.22 (−5.99 to 1.56)	−2.35 (−6.01 to 1.32)	0.715
Blood glucose, mg/dL	−1.15 (−1.83 to −0.48)†	−1.21 (−1.90 to −0.52)†	−0.92 (−1.58 to −0.26)†	−0.84 (−1.51 to −0.18)*	−1.25 (−1.91 to −0.59)‡	0.781
Hemoglobin A1c, %	−0.01 (−0.02 to 0.00)	−0.01 (−0.03 to 0.00)	−0.02 (−0.03 to −0.01)†	−0.02 (−0.04 to −0.01)†	−0.02 (−0.04 to −0.01)‡	0.119
Salt Check Sheet score	−0.24 (−0.41 to −0.07)†	−0.24 (−0.42 to −0.06)†	−0.29 (−0.46 to −0.12)†	−0.27 (−0.44 to −0.09)†	−0.28 (−0.45 to −0.11)†	0.598

*BP* blood pressure, *CI* confidence interval, *HDL* high-density lipoprotein, *K* potassium, *LDL* low-density lipoprotein, *Na/K ratio* sodium-to-potassium ratio

Mean differences in changes from baseline to follow-up for endpoint parameters in the intensive support programs vs. the standard support group (*N* = 3585) adjusted for age, sex, body mass index, smoking status, alcohol drinking, exercise, hemoglobin A1c, medication for hypertension, diabetes, and dyslipidemia, history of stroke, heart disease, and chronic kidney disease, and study field type. Further adjusted for the timing of the urinary test for the analysis of urinary indices

*P* values: *<0.05, †<0.01, and ‡<0.001

### Sensitivity analyses by demographics, lifestyle factors, and medical history

Supplementary Tables 3 to 12 present the results of sensitivity analyses stratified by baseline demographics, lifestyle factors, and medical history. For the primary endpoints, more negative between-group mean differences in the urinary Na/K ratio and estimated salt intake, and more positive mean differences in the estimated K intake, were observed in the intensive support group than in the standard support group, particularly among participants aged ≥65 years, those with a higher estimated K intake, or those with BMI < 25 kg/m² (Supplementary Tables 4, 7, 9). For the secondary endpoints, heterogeneities were noted in certain subgroups. The intensive support group exhibited more negative mean differences in systolic BP, especially among participants aged ≥65 years, those with a higher estimated salt intake, and those with prevalent hypertension (Supplementary Tables 4, 6, and 10). In contrast, more positive mean differences in diastolic BP were observed among men and participants from workplaces (Supplementary Tables 3 and 12). Furthermore, the intensive support group showed more negative mean differences in blood glucose among men, participants aged 18 to 64 years, and participants from workplaces (Supplementary Tables 3, 4, 12). In the analyses stratified by urinary sampling timing (Supplementary Table 13), between-group differences in the urinary Na/K ratio and in the estimated K intake were generally consistent across sampling timings. Although a statistically significant heterogeneity was observed for estimated salt intake (P for heterogeneity = 0.033), the overall between-group difference in the estimated salt intake was not statistically significant.

## Discussion

In this analysis of multicenter population-approach programs for salt reduction and increased K intake, 1-year intensive support programs across populations were associated with a decreased urinary Na/K ratio and a smaller decline in the estimated K intake compared with those of the standard support program, although no significant difference was observed in the estimated salt intake. The intensive support programs were also associated with improvements, including smaller increases in blood glucose, reductions in hemoglobin A1c and Salt Check Sheet scores, and an increase in HDL cholesterol levels. The mean differences in the changes in endpoint parameters between the intensive and standard programs were particularly pronounced in specific subgroups, with greater between-group differences in the urinary Na/K ratio, estimated salt intake, and estimated K intake observed particularly among older participants, those without obesity, or those with a higher K intake.

We evaluated the association of comprehensive intensive support programs aimed at reducing salt intake and increasing K intake, which included on-site urinary Na/K ratio measurement with immediate feedback, the use of textbooks or smartphone-based messaging apps, repeated personalized health guidance, and repeated urinary Na/K ratio measurement, with changes in the urinary Na/K ratio, estimated Na and K intakes, and cardiovascular risk factors. The lack of statistically significant differences in the associations across different program types suggests that each type may have a relatively uniform impact. In addition, the food environment intervention included a common set of required items in all intensive program fields. These food environment improvements were considered to have contributed to the implementation and recall of the health guidance content, thereby supporting the uniform impact of the program types. Improving lifestyle habits aimed at salt reduction and increased K intake for BP management may have also contributed to the increase in HDL cholesterol and reductions in diabetes markers. Our findings partially support previous studies that demonstrated the benefits of monitoring urinary Na excretion or the Na/K ratio [10, 20], app-based interventions [21], or personalized health guidance [22] in promoting reduced Na intake or improved K intake.

The observed effect size, with a mean reduction in the urinary Na/K ratio of 0.14 and a between-group difference corresponding to a smaller decline in estimated K intake of approximately 31 mg/day, was modest. Similar previous studies are limited; however, a 3-month randomized trial among healthy adults found that an information and communication technology-based online education intervention led to a modest reduction of 0.9 (95% CI, 0.0 to 1.8) in the spot urinary Na/K ratio compared with that in the control group [23]. In the National Health and Nutrition Examination Survey in the US, a 0.5-unit higher urinary Na/K ratio was associated with a 1.72 mmHg higher systolic BP, and each 1000 mg/day higher K intake was associated with a 3.72 mmHg lower systolic BP [24]. Under these graded associations, the −0.14-unit reduction in urinary Na/K ratio observed in our study would correspond to approximately 0.5 mmHg lower systolic BP, and the +31 mg/day difference in estimated K intake would correspond to approximately 0.1 mmHg difference in systolic BP. However, implementing such intensive support programs on a large scale, such as nationally (e.g., through the nationwide health check-up and guidance), could offer significant public health benefits [25, 26]. Moreover, it has been estimated that a 1-mmHg population-wide reduction in systolic BP is associated with a substantial reduction in CVD incidence [27]. Similarly, even small changes in the urinary Na/K ratio and K intake might contribute to improved BP management across populations [28]. In this context, recent perspectives have emphasized that improving K adequacy is an important and scalable component of dietary strategies for population BP control [29]. Furthermore, the average K intake in our study population (~2000 mg/day) remains below the Japanese Dietary Reference Intakes (3000 mg/day for men and 2600 mg/day for women) [30], suggesting that incremental improvements may represent realistic progress toward recommended intake targets.

The observed difference in the change in the urinary Na/K ratio may be attributed to the difference in the change in the estimated K intake between the intensive and standard support programs. In contrast, no difference was observed in the estimated salt intake change between the programs. In our study, the intensive support programs recommended reducing salt intake while increasing K consumption. However, in the context of the Japanese diet, K and salt intakes tend to be positively correlated, as higher vegetable consumption, which is rich in K, often accompanies higher salt intake [31]. The positive between-group mean differences in estimated K intake associated with the intensive support programs, together with the absence of a corresponding between-group difference in estimated salt intake, may reflect the success of efforts to reduce salt consumption while promoting K-rich dietary choices.

The intensive support group showed a greater increase in diastolic BP than the standard support group in the overall analysis. This may be explained by the pronounced increases in diastolic BP observed in specific subgroups, such as men and participants from certain workplaces (Supplementary Tables 3 and 12). One possible explanation is the lack of adequate standardization in BP measurement methods between 2021 and 2022 within workplaces. In one workplace within the intensive support group, systolic and diastolic BP increased markedly from 2021 to 2022 (7.53 mmHg and 7.01 mmHg, respectively; Supplementary Table 2). In contrast, the intensive support group exhibited more negative between-group mean differences in the urinary Na/K ratio and estimated salt intake, and more positive mean differences in estimated K intake, along with more negative between-group mean differences in systolic BP compared with the standard support group, particularly among older participants, those with higher salt intake, and those with prevalent hypertension (Supplementary Tables 4, 6, 10). These findings suggest that the intensive support programs may be particularly effective at lowering BP through salt reduction and improved K intake in populations susceptible to high BP. A modest heterogeneity was observed for estimated salt intake according to urine sampling timing. This may reflect the opposite directions of the between-group differences across sampling timings. However, because the overall between-group difference in estimated salt intake was not statistically significant, this finding should be interpreted with caution.

Several limitations of our study warrant consideration. First, the retrospective observational design limits causal inferences, despite adjustments for key confounders, and selection bias may have influenced our findings. Additionally, despite multivariable adjustments, residual confounding from unmeasured variables cannot be excluded. Second, urinary Na and K were measured using casual urine samples, and estimated salt and K intakes were calculated using the Tanaka formula. Casual urine may not accurately reflect 24-h urinary excretion because of intra-individual variability, and potential biases may exist with the formula [32]. However, because of the labor-intensive and time-consuming nature of 24-h collections, single casual urine samples would be practical to evaluate the population level of the urinary Na/K ratio. Third, the nonstandardized measurement procedures of endpoints and covariates across study fields, including differences in BP measurement frequency, might have introduced potential bias into the findings. Fourth, data on total energy intake and biochemical markers of nutritional status, such as plasma albumin levels, were not available in this study. Therefore, we cannot exclude the possibility that changes in overall dietary intake or nutritional status may have influenced the observed associations. Fifth, the study population primarily included participants from municipalities and workplaces engaged in health programs, which may limit the generalizability of the findings to other populations, particularly those with different socioeconomic or geographic backgrounds. Sixth, adherence to the components of the intensive support programs, such as the use of smartphone-based messaging apps, repeated measurement of the urinary Na/K ratio, and engagement with health guidance materials, was not assessed, which could have influenced the observed outcomes. Finally, the study’s 1-year follow-up period may not fully capture the long-term sustainability of the observed effects or their impact on clinical outcomes such as cardiovascular events.

### Perspective of Asia

In many Asian countries, dietary patterns are characterized by high Na intake and insufficient K intake, largely influenced by traditional food cultures, making sustained dietary modification challenging in real-world settings [33, 34]. In this context, individual-level approaches alone may be insufficient, and population-based strategies are increasingly recognized as essential. Japan represents a typical example, where Na intake remains above recommended levels and K intake is suboptimal [30]. Our findings suggest that population-approach programs promoting Na reduction and K intake implemented across municipalities and workplaces can achieve modest but measurable improvements in dietary-related indicators, particularly the urinary Na/K ratio. Although the effect sizes were modest, such programs may contribute to meaningful shifts in population BP distribution [25, 26], underscoring the importance of population-based approaches for hypertension prevention and management in Asia.

## Conclusions

Our study demonstrated that comprehensive, 1-year intensive support programs involving a population approach promoting salt reduction and increased K intake across populations were associated with a reduction in the urinary Na/K ratio and a positive between-group difference in estimated K intake, as well as improvements in several cardiovascular risk factors. While the effect size may be modest, these findings suggest that such population-approach programs have the potential to improve dietary behaviors and cardiovascular risk profiles. Our findings may contribute to the development of effective hypertension prevention and management strategies aimed at promoting healthier dietary behaviors, such as reduced salt intake and improved K intake, which can be implemented in community and workplace settings, offering wide applicability in public health promotion efforts.

## Supplementary information

Supplementary information

## Data Availability

Data are available from the corresponding authors upon reasonable request.

## References

[1] Hisamatsu T, Segawa H, Kadota A, Ohkubo T, Arima H, Miura K. Epidemiology of hypertension in Japan: beyond the new 2019 Japanese guidelines. Hypertens Res. 2020;43:1344–51. 32636526 10.1038/s41440-020-0508-z

[2] Weinberger MH. Sodium, potassium, and blood pressure. Am J Hypertens. 1997;10:46S–8S. 9160780

[3] Hisamatsu T, Lloyd-Jones DM, Colangelo LA, Liu K. Urinary sodium and potassium excretions in young adulthood and blood pressure by middle age: the Coronary Artery Risk Development in Young Adults (CARDIA) Study. J Hypertens. 2021;39:1586–93. 34188003 10.1097/HJH.0000000000002802

[4] He FJ, MacGregor GA. Effect of longer-term modest salt reduction on blood pressure. Cochrane Database Syst Rev. 2004;CD004937. 10.1002/14651858.CD004937 15266549

[5] Hisamatsu T, Kogure M, Tabara Y, Hozawa A, Sakima A, Tsuchihashi T, et al. Practical use and target value of urine sodium-to-potassium ratio in assessment of hypertension risk for Japanese: Consensus Statement by the Japanese Society of Hypertension Working Group on Urine Sodium-to-Potassium Ratio. Hypertens Res. 2024;47:3288–302. 39375509 10.1038/s41440-024-01861-x

[6] Tabara Y, Takahashi Y, Kumagai K, Setoh K, Kawaguchi T, Takahashi M, et al. Descriptive epidemiology of spot urine sodium-to-potassium ratio clarified a close relationship with blood pressure level: the Nagahama study. J Hypertens. 2015;33:2407–13. 26378682 10.1097/HJH.0000000000000734

[7] Ministry of Health, Labour and Welfare. Explanation Materials for the Promotion of Health Japan 21 (Third Term) (in Japanese). https://www.mhlw.go.jp/stf/seisakunitsuite/bunya/kenkou_iryou/kenkou/kenkounippon21_00006.html (accessed December 20, 2024).

[8] Santos JA, Tekle D, Rosewarne E, Flexner N, Cobb L, Al-Jawaldeh A, et al. A Systematic Review of Salt Reduction Initiatives Around the World: A Midterm Evaluation of Progress Towards the 2025 Global Non-Communicable Diseases Salt Reduction Target. Adv Nutr. 2021;12:1768–80. 33693460 10.1093/advances/nmab008 PMC8483946

[9] Ministry of Health, Labour and Welfare. Large-scale Demonstration Project on Prevention and Health Promotion (in Japanese). https://www.mhlw.go.jp/stf/seisakunitsuite/bunya/0000120172_00014.html (accessed November 25, 2025).

[10] Iwahori T, Ueshima H, Ohgami N, Yamashita H, Miyagawa N, Kondo K, et al. Effectiveness of a self-monitoring device for urinary sodium-to-potassium ratio on dietary improvement in free-living adults: a randomized controlled trial. J Epidemiol. 2018;28:41–7. 29093302 10.2188/jea.JE20160144 PMC5742378

[11] Yatabe J, Ishida K, Yatabe MS. Old story, new twist: reducing salt and increasing potassium intake as a social issue according to the INTERMAP Japan. Hypertens Res. 2023;46:526–8. 36357617 10.1038/s41440-022-01082-0

[12] Ministry of Health, Labour and Welfare. The Report of the Working Group on Food Environment Improvement for Health Promotion (in Japanese). https://www.mhlw.go.jp/shingi/2004/12/s1202-4.html (accessed December 20, 2024).

[13] Hayabuchi H, Takemi Y, Ohta M, Sakata I, Sakaguchi K, Kubo A, et al. Development of a new method for assessing the availability of low-sodium foods in Japan. Nihon Koshu Eisei Zasshi. 2024;71:366–75. 38556361 10.11236/jph.23-094

[14] Tanaka T, Okamura T, Miura K, Kadowaki T, Ueshima H, Nakagawa H, et al. A simple method to estimate population 24-h urinary sodium and potassium excretion using a casual urine specimen. J Hum Hypertens. 2002;16:97–103. 11850766 10.1038/sj.jhh.1001307

[15] Yasutake K, Miyoshi E, Kajiyama T, Umeki Y, Misumi Y, Horita N, et al. Comparison of a salt check sheet with 24-h urinary salt excretion measurement in local residents. Hypertens Res. 2016;39:879–85. 27383507 10.1038/hr.2016.79

[16] Miyachi M. Measures of physical activity and exercise for health promotion by the Ministry of Health, Labour and Welfare. J Phys Fit Sports Med. 2012;1:467–72.

[17] Ohya Y, Arakawa K, Arata N, Arima S, Arima H, Asayama K, et al. The Japanese Society of Hypertension Guidelines for the management of elevated blood pressure and hypertension 2025 (JSH2025). Hypertens Res. 2026;49:9–235. 41476123 10.1038/s41440-025-02462-y

[18] Okamura T, Tsukamoto K, Arai H, Fujioka Y, Ishigaki Y, Koba S, et al. Japan Atherosclerosis Society (JAS) Guidelines for Prevention of Atherosclerotic Cardiovascular Diseases 2022. J Atheroscler Thromb. 2023;31:641–853. 38123343 10.5551/jat.GL2022 PMC11150976

[19] Araki E, Goto A, Kondo T, Noda M, Noto H, Origasa H, et al. Japanese Clinical Practice Guideline for Diabetes 2019. J Diabetes Investig. 2020;11:1020–76. 33021749 10.1111/jdi.13306 PMC7378414

[20] Takada T, Imamoto M, Sasaki S, Azuma T, Miyashita J, Hayashi M, et al. Effects of self-monitoring of daily salt intake estimated by a simple electrical device for salt reduction: a cluster randomized trial. Hypertens Res. 2018;41:524–30. 29695772 10.1038/s41440-018-0046-0

[21] He FJ, Zhang P, Luo R, Li Y, Sun Y, Chen F, et al. App-based education programme to reduce salt intake (AppSalt) in schoolchildren and their families in China: parallel, cluster randomised controlled trial. BMJ. 2022;376:e066982. 35140061 10.1136/bmj-2021-066982 PMC8826455

[22] Livingstone KM, Celis-Morales C, Navas-Carretero S, San-Cristobal R, Forster H, Woolhead C, et al. Personalised nutrition advice reduces intake of discretionary foods and beverages: findings from the Food4Me randomised controlled trial. Int J Behav Nutr Phys Act. 2021;18:70. 34092234 10.1186/s12966-021-01136-5 PMC8183081

[23] Yano Y, Kitaoka K, Ohkubo T, Okamura T, Kanegae H, Yoshita K, et al. A randomized clinical trial of ICT-based interventions for sodium and potassium regulation in healthy adults. Am J Hypertens. 2025;38:588–94. 40214261 10.1093/ajh/hpaf049 PMC12260158

[24] Jackson SL, Cogswell ME, Zhao L, Terry AL, Wang CY, Wright J, et al. Association between urinary sodium and potassium excretion and blood pressure among adults in the United States: National Health and Nutrition Examination Survey, 2014. Circulation. 2018;137:237–46. 29021321 10.1161/CIRCULATIONAHA.117.029193 PMC5771856

[25] Rose G. Sick individuals and sick populations. Int J Epidemiol. 2001;30:427–32. 11416056 10.1093/ije/30.3.427

[26] Rose G. The strategy of preventive medicine. New edn. Oxford University Press: Oxford; 1993.

[27] Hardy ST, Loehr LR, Butler KR, Chakladar S, Chang PP, Folsom AR, et al. Reducing the blood pressure-related burden of cardiovascular disease: impact of achievable improvements in blood pressure prevention and control. J Am Heart Assoc. 2015;4:e002276. 26508742 10.1161/JAHA.115.002276 PMC4845128

[28] Stamler R. Implications of the INTERSALT study. Hypertension. 1991;17:I16–20. 1986996 10.1161/01.hyp.17.1_suppl.i16

[29] Chan RJ, Parikh N, Ahmed S, Ruzicka M, Hiremath S. Blood pressure control should focus on more potassium: controversies in hypertension. Hypertension. 2024;81:501–9. 37641923 10.1161/HYPERTENSIONAHA.123.20545

[30] Ministry of Health, Labour and Welfare. Dietary reference intakes for Japanese, 2025 (in Japanese). https://www.mhlw.go.jp/content/10904750/001316585.pdf (accessed March 3, 2026).

[31] Kondo K, Miura K, Okamura T, Okayama A, Ueshima H. Dietary factors, dietary patterns, and cardiovascular disease risk in representative Japanese Cohorts: NIPPON DATA80/90. J Atheroscler Thromb. 2023;30:207–19. 36436878 10.5551/jat.RV22001 PMC9981349

[32] Campbell NRC, Whelton PK, Orias M, Cobb LL, Jones ESW, Garg R, et al. It is strongly recommended not to conduct, fund, or publish research studies that use spot urine samples with estimating equations to assess individuals’ sodium (salt) intake in association with health outcomes: a policy statement of the World Hypertension League, International Society of Hypertension and Resolve to Save Lives. J Hypertens. 2023;41:683–6. 36723484 10.1097/HJH.0000000000003385 PMC10090307

[33] Powles J, Fahimi S, Micha R, Khatibzadeh S, Shi P, Ezzati M, et al. Global, regional and national sodium intakes in 1990 and 2010: a systematic analysis of 24 h urinary sodium excretion and dietary surveys worldwide. BMJ Open. 2013;3:e003733. 24366578 10.1136/bmjopen-2013-003733 PMC3884590

[34] Reddin C, Ferguson J, Murphy R, Clarke A, Judge C, Griffith V, et al. Global mean potassium intake: a systematic review and Bayesian meta-analysis. Eur J Nutr. 2023;62:2027–37. 36882596 10.1007/s00394-023-03128-6 PMC10349712

