# Supplementary material for: Effect of population-approach programs promoting salt reduction and potassium intake in Japan: the Population-based Sodium/Potassium Improvement Program (PoSPIP)
Source: Hypertens Res. 2026 May 14;49(7):1992–2003. doi: 10.1038/s41440-026-02662-0 (PMC13333495; doi:10.1038/s41440-026-02662-0)
Supplement: Supplementary file 1 — Supplementary information [file 41440_2026_2662_MOESM1_ESM.docx]

**SUPPLEMENTARY INFORMATION**

**Effect of population-approach programs promoting salt reduction and potassium intake in Japan: The Population-based Sodium/Potassium Improvement Program (PoSPIP)**

Takashi Hisamatsu, MD, PhD,^1^ Minako Kinuta, PhD,^1,2^ Takayoshi Ohkubo, MD, PhD,^3^ Takuya Tsuchihashi, MD, PhD,^4^ Katsushi Yoshita, PhD,^5^ Yukari Takemi, PhD,^6^ Hitomi Hayabuchi, PhD,^7^ Yukiko Okami, PhD,^8,9^ Kaori Kitaoka, PhD,^9,10^ Keiko Sakaguchi, PhD,^11^ Atsushi Hozawa, MD, PhD,^12^ Tomonori Okamura, MD, PhD,^13^ Hiroshi Itoh, MD, PhD,^14,15^ Hiromi Rakugi MD, PhD,^14,16^ Koichi Node, MD, PhD,^14,17^ Katsuyuki Miura, MD, PhD,^9,14^ for the PoSPIP Research Group

^1^Department of Public Health, Okayama University Graduate School of Medicine, Dentistry and Pharmaceutical Sciences, Okayama, Japan

^2^Department of Food Sciences and Nutrition, Mukogawa Women’s University, Nishinomiya, Japan

^3^Department of Hygiene and Public Health, Teikyo University School of Medicine, Tokyo, Japan

^4^Cardiovascular Center, Steel Memorial Yawata Hospital, Kitakyushu, Japan

^5^Department of Public Health, Nutrition, School of Human Life and Ecology, Osaka Metropolitan University, Osaka, Japan

^6^Faculty of Nutrition, Kagawa Nutrition University, Sakado, Japan

^7^Graduate School of Health and Environmental Sciences, Fukuoka Women’s University, Fukuoka, Japan

^8^Gunma University Center for Food Science and Wellness, Maebashi, Japan

^9^NCD Epidemiology Research Center, Shiga University of Medical Science, Otsu, Japan

^10^Section of Research Collaboration and Partnership, Center for Private-Public-Academic Collaboration Research, National Institutes of Biomedical Innovation, Health and Nutrition, Settsu, Japan

^11^Faculty of Nursing and Nutrition, Shukutoku University, Chiba, Japan

^12^Division of Epidemiology, School of Public Health, Tohoku University Graduate School of Medicine, Sendai, Japan

^13^Department of Preventive Medicine and Public Health, Keio University School of Medicine, Tokyo, Japan

^14^Japanese Society of Hypertension, Tokyo, Japan

^15^The Center for Preventive Medicine, Keio University, Tokyo, Japan

^16^Osaka Rosai Hospital, Sakai, Japan

^17^Department of Cardiovascular Medicine, Saga University, Saga, Japan

**Supplementary Table 1.** Details of the initiatives for food environment improvements in the municipalities.

| Dimension of initiatives | Required or voluntary | Items carried out |
| --- | --- | --- |
| Food availability/ accessibility | required | Assessment of the availability of low-salt foods. |
|  | required | Improved the availability of low-salt foods. |
|  | voluntary | Sales of foods with salt intake reduction and increased potassium intake, with POP postings. |
|  | voluntary | Certification of nutritionally balanced diet called Smart Meal. |
|  | voluntary | Development of low-salt foods by local food enterprises. |
|  | voluntary | Community-specific initiatives. |
| Information availability/ accessibility | required | Project page on the municipality's website. |
|  | required | Providing information on the availability of JSH low-salt foods. |
|  | required | Compiling and publishing a list of low-salty foods sold in the region. |
|  | required | POP displays salt reduction and potassium increase. |
|  | voluntary | Use of municipal public relations |
|  | voluntary | Use of local mass media. For example, cable television etc. |
|  | voluntary | Community-specific initiatives. |
| Create a momentum | required | Salt Reduction Day (17th of each month) or Food and Nutrition Education Day (19th of each month) campaign. |
|  | voluntary | Installation of banners and hanging banners. |
|  | voluntary | Declaration by the Mayor in relation to the project. |
|  | voluntary | Use of project materials by local councilors. |
|  | voluntary | Creating a system of competition for the efforts of health guidance targets. |
|  | voluntary | Community-specific initiatives. |

Similar items were carried out in the workplaces. JSH, Japan Society of Hypertension; POP, point of purchase

**Supplementary Table 2**. The distributions of demographic and primary and secondary endpoint parameters of participants in municipalities and workplaces.

| Intensive support group | Municipalities | | | | | | | |
| --- | --- | --- | --- | --- | --- | --- | --- | --- |
|  | A, n=213 | | B, n=337 | | C, n=324 | | D, n=678 | |
|  | Baseline | Follow-up | Baseline | Follow-up | Baseline | Follow-up | Baseline | Follow-up |
| Age, years | 64.9 (8.1) |  | 60.8 (12.8) |  | 66.6 (6.9) |  | 63.1 (11.0) |  |
| Women, n (%) | 128 (60.1) |  | 166 (49.3) |  | 191 (59.0) |  | 439 (64.8) |  |
| Primary endpoints |  |  |  |  |  |  |  |  |
| Urinary Na/K ratio | 4.48 (2.23) | 4.14 (2.45) | 2.94 (1.92) | 2.98 (2.11) | 4.36 (2.66) | 4.34 (2.61) | 4.50 (2.58) | 4.43 (2.47) |
| Estimated daily salt intake, g/day | 8.88 (2.16) | 8.59 (1.88) | 8.87 (2.24) | 9.00 (2.39) | 8.74 (1.85) | 8.78 (1.83) | 8.97 (2.00) | 8.89 (1.99) |
| Estimated daily K intake, mg/day | 1760 (340) | 1784 (358) | 2126 (389) | 2155 (420) | 1795 (355) | 1802 (358) | 1805 (361) | 1803 (381) |
| Secondary endpoints |  |  |  |  |  |  |  |  |
| Systolic BP, mmHg | 134.07 (15.76) | 133.97 (15.59) | 129.78 (15.93) | 130.06 (15.17) | 124.43 (17.28) | 123.41 (17.36) | 127.02 (19.63) | 123.79 (18.08) |
| Diastolic BP, mmHg | 79.95 (10.04) | 79.85 (9.26) | 79.76 (10.20) | 79.34 (9.78) | 75.38 (11.12) | 74.27 (11.45) | 77.05 (11.35) | 75.56 (10.79) |
| Body weight, kg | 60.82 (12.25) | 60.64 (12.19) | 63.58 (14.41) | 63.14 (14.46) | 58.14 (11.11) | 57.96 (11.33) | 57.31 (10.57) | 56.91 (10.53) |
| LDL cholesterol, mg/dL | 128.61 (33.43) | 122.36 (30.27) | 120.87 (33.54) | 117.06 (32.43) | 129.81 (30.48) | 124.79 (29.17) | 125.53 (30.27) | 122.72 (29.91) |
| HDL cholesterol, mg/dL | 64.58 (17.56) | 65.03 (18.45) | 64.80 (16.71) | 65.50 (17.38) | 68.11 (18.72) | 68.39 (18.93) | 71.02 (18.45) | 71.06 (18.38) |
| Triglycerides, mg/dL | 119.89 (96.55) | 116.83 (92.88) | 123.71 (89.84) | 117.16 (75.41) | 101.40 (62.99) | 102.32 (53.70) | 102.18 (54.31) | 102.91 (59.39) |
| Blood glucose, mg/dL | 103.10 (18.04) | 102.48 (17.25) | 103.01 (21.06) | 103.61 (20.83) | 95.84 (15.09) | 97.35 (15.20) | 99.60 (16.17) | 99.38 (15.41) |
| Hemoglobin A1c, % | 5.82 (0.68) | 5.84 (0.62) | 5.63 (0.68) | 5.66 (0.70) | 5.53 (0.55) | 5.53 (0.56) | 5.55 (0.53) | 5.51 (0.55) |
| Salt check sheet score | 12.35 (3.97) | 11.82 (3.96) | 13.36 (5.05) | 12.71 (4.28) | 10.92 (4.01) | 10.48 (4.07) | 11.40 (4.12) | 10.58 (4.04) |

In accordance with the contract with the Ministry of Health, Labour and Welfare, the names of municipalities and workplaces are kept anonymous. Values are expressed as the means (standard deviations) or numbers (percentages). BP, blood pressure; HDL, high-density lipoprotein; K, potassium; LDL, low-density lipoprotein; Na/K ratio, sodium-to-potassium ratio.

**Supplementary Table 2 (continued)**. The distributions of demographic and primary and secondary endpoint parameters of participants in municipalities and workplaces.

| Intensive support group | Municipalities | | | | | |
| --- | --- | --- | --- | --- | --- | --- |
|  | E, n=218 | | F, n=465 | | G, n=668 | |
|  | Baseline | Follow-up | Baseline | Follow-up | Baseline | Follow-up |
| Age, years | 67.7 (5.9) |  | 63.8 (10.2) |  | 66.0 (7.1) |  |
| Women, n (%) | 122 (56.0) |  | 262 (56.3) |  | 365 (54.6) |  |
| Primary endpoints |  |  |  |  |  |  |
| Urinary Na/K ratio | 4.05 (2.39) | 4.06 (2.44) | 4.88 (2.60) | 4.97 (2.95) | 2.33 (1.31) | 2.32 (1.29) |
| Estimated daily salt intake, g/day | 9.15 (2.10) | 9.14 (2.06) | 9.35 (2.04) | 9.31 (2.12) | 8.28 (1.89) | 8.04 (1.91) |
| Estimated daily K intake, mg/day | 1943 (408) | 1954 (398) | 1818 (385) | 1806 (388) | 2182 (380) | 2111 (368) |
| Secondary endpoints |  |  |  |  |  |  |
| Systolic BP, mmHg | 134.49 (16.55) | 131.55 (16.11) | 127.42 (17.47) | 125.91 (15.85) | 123.30 (14.95) | 124.18 (15.35) |
| Diastolic BP, mmHg | 77.07 (10.01) | 75.70 (10.04) | 74.22 (10.03) | 75.68 (10.76) | 72.26 (9.36) | 72.94 (9.42) |
| Body weight, kg | 57.72 (10.52) | 57.18 (10.28) | 58.11 (11.03) | 57.76 (11.01) | 57.40 (11.36) | 57.11 (11.36) |
| LDL cholesterol, mg/dL | 130.84 (29.60) | 122.61 (30.99) | 114.71 (25.90) | 119.75 (27.41) | 123.20 (27.85) | 120.27 (26.54) |
| HDL cholesterol, mg/dL | 62.45 (16.09) | 65.03 (16.84) | 62.58 (15.41) | 64.42 (15.16) | 64.34 (17.20) | 66.73 (17.92) |
| Triglycerides, mg/dL | 132.96 (82.80) | 126.36 (81.80) | 106.33 (69.48) | 107.63 (76.88) | 100.49 (54.40) | 99.40 (51.57) |
| Blood glucose, mg/dL | 97.85 (13.25) | 100.69 (23.49) | 99.85 (17.96) | 100.28 (17.00) | 101.12 (17.16) | 101.74 (14.23) |
| Hemoglobin A1c, % | 5.79 (0.68) | 5.88 (0.74) | 5.67 (0.68) | 5.62 (0.63) | 5.66 (0.50) | 5.67 (0.48) |
| Salt check sheet score | 10.93 (4.02) | 10.30 (4.06) | 12.49 (4.02) | 11.69 (4.29) | 12.13 (4.42) | 11.56 (4.34) |

In accordance with the contract with the Ministry of Health, Labour and Welfare, the names of municipalities and workplaces are kept anonymous. Values are expressed as the means (standard deviations) or numbers (percentages). BP, blood pressure; HDL, high-density lipoprotein; K, potassium; LDL, low-density lipoprotein; Na/K ratio, sodium-to-potassium ratio.

**Supplementary Table 2 (continued)**. The distributions of demographic and primary and secondary endpoint parameters of participants in municipalities and workplaces.

| Intensive support group | Workplaces | | | | | |
| --- | --- | --- | --- | --- | --- | --- |
|  | H, n=938 | | I, n=113 | | J, n=110 | |
|  | Baseline | Follow-up | Baseline | Follow-up | Baseline | Follow-up |
| Age, years | 37.5 (12.7) |  | 43.1 (10.6) |  | 49.4 (9.4) |  |
| Women, n (%) | 234 (25.0) |  | 14 (12.4) |  | 9 (8.2) |  |
| Primary endpoints |  |  |  |  |  |  |
| Urinary Na/K ratio | 3.03 (1.94) | 3.02 (1.95) | 4.16 (2.23) | 4.07 (2.47) | 5.98 (5.27) | 5.87 (3.93) |
| Estimated daily salt intake, g/day | 8.96 (2.02) | 8.94 (2.11) | 8.14 (1.66) | 8.20 (1.82) | 8.89 (2.31) | 9.05 (2.32) |
| Estimated daily K intake, mg/day | 2163 (445) | 2153 (429) | 1698 (433) | 1726 (449) | 1679 (407) | 1686 (398) |
| Secondary endpoints |  |  |  |  |  |  |
| Systolic BP, mmHg | 117.68 (14.54) | 118.63 (14.84) | 108.89 (12.48) | 116.42 (13.41) | 127.05 (12.94) | 124.05 (10.78) |
| Diastolic BP, mmHg | 72.97 (11.23) | 74.75 (11.31) | 68.27 (9.31) | 75.28 (10.00) | 79.05 (9.18) | 78.15 (9.23) |
| Body weight, kg | 65.74 (12.92) | 66.28 (13.19) | 64.76 (10.82) | 64.93 (10.95) | 69.70 (15.32) | 69.30 (15.46) |
| LDL cholesterol, mg/dL | 118.94 (30.93) | 118.77 (30.75) | 123.68 (30.50) | 125.04 (30.69) | 128.40 (30.48) | 118.69 (30.30) |
| HDL cholesterol, mg/dL | 61.76 (14.69) | 60.97 (14.02) | 63.73 (15.37) | 65.65 (15.90) | 59.09 (16.41) | 59.37 (16.49) |
| Triglycerides, mg/dL | 109.61 (82.13) | 101.54 (69.15) | 89.07 (57.34) | 86.23 (56.07) | 107.65 (64.11) | 109.80 (72.54) |
| Blood glucose, mg/dL | 93.30 (13.92) | 92.11 (16.45) | 89.89 (8.12) | 88.68 (8.81) | 102.30 (23.13) | 101.04 (15.60) |
| Hemoglobin A1c, % | 5.33 (0.46) | 5.25 (0.47) | 5.35 (0.45) | 5.33 (0.46) | 5.58 (0.79) | 5.56 (0.52) |
| Salt check sheet score | 13.54 (4.12) | 13.00 (4.28) | 12.80 (3.52) | 12.74 (3.84) | 13.47 (4.35) | 12.34 (4.59) |

In accordance with the contract with the Ministry of Health, Labour and Welfare, the names of municipalities and workplaces are kept anonymous. Values are expressed as the means (standard deviations) or numbers (percentages). BP, blood pressure; HDL, high-density lipoprotein; K, potassium; LDL, low-density lipoprotein; Na/K ratio, sodium-to-potassium ratio.

**Supplementary Table 2 (continued)**. The distributions of demographic and primary and secondary endpoint parameters of participants in municipalities and workplaces.

| Standard support group | Municipalities | | | | | | | |
| --- | --- | --- | --- | --- | --- | --- | --- | --- |
|  | K, n=217 | | L, n=565 | | M, n=162 | | N, n=34 | |
|  | Baseline | Follow-up | Baseline | Follow-up | Baseline | Follow-up | Baseline | Follow-up |
| Age, years | 64.0 (9.3) |  | 66.4 (7.3) |  | 64.7 (8.7) |  | 63.8 (8.6) |  |
| Women, n (%) | 120 (55.3) |  | 316 (55.9) |  | 80 (49.4) |  | 22 (64.7) |  |
| Primary endpoints |  |  |  |  |  |  |  |  |
| Urinary Na/K ratio | 4.06 (2.18) | 4.01 (2.00) | 4.32 (2.59) | 4.18 (2.57) | 4.76 (3.79) | 4.33 (2.83) | 4.12 (1.96) | 4.50 (3.24) |
| Estimated daily salt intake, g/day | 8.73 (2.16) | 8.93 (2.01) | 8.75 (1.89) | 8.66 (1.81) | 9.22 (2.13) | 9.19 (2.27) | 8.66 (2.17) | 8.54 (1.92) |
| Estimated daily K intake, mg/day | 1834 (453) | 1856 (326) | 1793 (345) | 1793 (344) | 1860 (459) | 1898 (426) | 1767 (390) | 1738 (445) |
| Secondary endpoints |  |  |  |  |  |  |  |  |
| Systolic BP, mmHg | 127.64 (17.26) | 127.67 (18.16) | 125.79 (17.65) | 123.89 (16.55) | 127.92 (16.06) | 126.46 (14.85) | 130.74 (15.91) | 130.59 (21.63) |
| Diastolic BP, mmHg | 78.16 (10.45) | 77.75 (10.42) | 76.24 (11.04) | 74.92 (10.75) | 75.48 (10.66) | 73.22 (9.93) | 76.32 (8.01) | 74.18 (12.00) |
| Body weight, kg | 59.30 (11.52) | 59.48 (11.55) | 58.43 (11.27) | 58.16 (11.03) | 59.10 (12.23) | 59.01 (12.51) | 56.28 (10.77) | 55.42 (10.62) |
| LDL cholesterol, mg/dL | 121.54 (26.81) | 121.23 (26.72) | 130.06 (31.73) | 125.55 (31.04) | 135.38 (29.88) | 128.14 (31.12) | 138.71 (31.15) | 130.56 (32.67) |
| HDL cholesterol, mg/dL | 67.79 (17.65) | 69.41 (17.63) | 68.72 (18.65) | 69.92 (18.63) | 64.27 (16.65) | 62.29 (15.98) | 65.91 (16.86) | 67.74 (15.93) |
| Triglycerides, mg/dL | 105.76 (59.07) | 98.66 (54.57) | 104.36 (59.99) | 100.70 (51.25) | 129.01 (89.25) | 136.33 (99.04) | 108.94 (84.26) | 85.76 (40.95) |
| Blood glucose, mg/dL | 95.03 (14.07) | 95.94 (20.24) | 98.95 (22.04) | 97.63 (17.03) | 98.67 (19.74) | 97.50 (20.50) | 105.41 (16.41) | 102.94 (17.15) |
| Hemoglobin A1c, % | 5.79 (0.46) | 5.94 (0.63) | 5.60 (0.69) | 5.59 (0.63) | 5.55 (0.64) | 5.58 (0.54) | 5.55 (0.64) | 5.69 (0.94) |
| Salt check sheet score | 11.80 (4.15) | 11.27 (3.90) | 10.96 (4.14) | 10.78 (4.15) | 11.81 (4.44) | 11.52 (4.25) | 11.68 (3.36) | 11.61 (3.73) |

In accordance with the contract with the Ministry of Health, Labour and Welfare, the names of municipalities and workplaces are kept anonymous. Values are expressed as the means (standard deviations) or numbers (percentages). BP, blood pressure; HDL, high-density lipoprotein; K, potassium; LDL, low-density lipoprotein; Na/K ratio, sodium-to-potassium ratio.

**Supplementary Table 2 (continued)**. The distributions of demographic and primary and secondary endpoint parameters of participants in municipalities and workplaces.

| Standard support group | Municipalities | | Workplaces | | | |
| --- | --- | --- | --- | --- | --- | --- |
|  | O, n=707 | | P, n=1,846 | | Q, n=54 | |
|  | Baseline | Follow-up | Baseline | Follow-up | Baseline | Follow-up |
| Age, years | 65.6 (7.3) |  | 36.7 (12.0) |  | 41.8 (7.7) |  |
| Women, n (%) | 365 (51.6) |  | 611 (33.1) |  | 20 (37.0) |  |
| Primary endpoints |  |  |  |  |  |  |
| Urinary Na/K ratio | 4.01 (2.66) | 4.63 (2.83) | 3.09 (1.95) | 3.12 (2.11) | 4.40 (2.27) | 4.25 (3.46) |
| Estimated daily salt intake, g/day | 9.02 (2.41) | 9.15 (2.26) | 8.97 (2.11) | 8.79 (2.12) | 8.33 (1.72) | 8.12 (1.86) |
| Estimated daily K intake, mg/day | 1946 (454) | 1859 (445) | 2139 (424) | 2095 (429) | 1687 (380) | 1721 (392) |
| Secondary endpoints |  |  |  |  |  |  |
| Systolic BP, mmHg | 128.40 (16.95) | 129.41 (16.27) | 116.35 (14.30) | 116.70 (14.89) | 120.96 (11.60) | 126.91 (15.45) |
| Diastolic BP, mmHg | 76.77 (11.32) | 77.52 (10.59) | 71.68 (11.81) | 72.90 (11.90) | 74.13 (9.23) | 76.72 (12.54) |
| Body weight, kg | 58.67 (11.00) | 58.66 (11.17) | 63.77 (12.71) | 63.99 (13.07) | 67.84 (14.73) | 68.40 (15.23) |
| LDL cholesterol, mg/dL | 122.99 (30.86) | 119.71 (30.34) | 116.17 (31.29) | 115.49 (30.29) | 129.02 (31.57) | 123.20 (29.60) |
| HDL cholesterol, mg/dL | 63.44 (17.11) | 64.41 (17.22) | 63.97 (15.36) | 62.91 (15.05) | 60.48 (13.91) | 58.72 (14.41) |
| Triglycerides, mg/dL | 110.71 (117.92) | 113.84 (104.18) | 103.20 (82.40) | 100.50 (80.12) | 89.72 (47.42) | 87.20 (48.76) |
| Blood glucose, mg/dL | 99.08 (17.76) | 100.18 (17.48) | 91.97 (17.90) | 93.86 (15.75) | 92.67 (11.17) | 91.26 (14.90) |
| Hemoglobin A1c, % | 5.75 (0.60) | 5.74 (0.52) | 5.27 (0.43) | 5.24 (0.44) | 5.24 (0.32) | 5.34 (0.46) |
| Salt check sheet score | 12.26 (4.23) | 12.00 (4.24) | 13.53 (4.15) | 13.07 (4.13) | 12.69 (3.73) | 13.33 (3.61) |

In accordance with the contract with the Ministry of Health, Labour and Welfare, the names of municipalities and workplaces are kept anonymous. Values are expressed as the means (standard deviations) or numbers (percentages). BP, blood pressure; HDL, high-density lipoprotein; K, potassium; LDL, low-density lipoprotein; Na/K ratio, sodium-to-potassium ratio.

**Supplementary Table 3**. Mean differences (95% confidence intervals) in changes in primary and secondary endpoint parameters between the intensive and standard support groups by sex.

|  | Sex | |  |
| --- | --- | --- | --- |
|  | Men | Women | P for heterogeneity |
| No. of participants in intensive/standard support groups | 2,134/2,051 | 1,930/1,534 |  |
| Primary endpoints |  |  |  |
| Urinary Na/K ratio | −0.14 (−0.31 to 0.04) | −0.14 (−0.33 to 0.05) | 0.950 |
| Estimated daily salt intake, g/day | 0.03 (−0.12 to 0.17) | 0.01 (−0.14 to 0.17) | 0.901 |
| Estimated daily K intake, mg/day | 24 (−3 to 51) | 45 (17 to 73)† | 0.472 |
| Secondary endpoints |  |  |  |
| Systolic BP, mmHg | 0.12 (−0.66 to 0.90) | −0.61 (−1.53 to 0.32) | 0.297 |
| Diastolic BP, mmHg | 1.06 (0.50 to 1.61)‡ | −0.24 (−0.83 to 0.36) | <0.001 |
| Body weight, kg | −0.06 (−0.22 to 0.11) | −0.07 (−0.21 to 0.06) | 0.707 |
| LDL cholesterol, mg/dL | 0.67 (−0.54 to 1.88) | 0.98 (−0.43 to 2.39) | 0.998 |
| HDL cholesterol, mg/dL | 0.53 (0.07 to 0.99)* | 0.24 (−0.29 to 0.77) | 0.958 |
| Triglycerides, mg/dL | −0.64 (−5.80 to 4.53) | −2.43 (−6.42 to 1.56) | 0.340 |
| Blood glucose, mg/dL | −1.87 (−2.87 to −0.87)‡ | −0.14 (−0.80 to 0.53) | 0.008 |
| Hemoglobin A1c, % | −0.04 (−0.06 to −0.02)‡ | −0.01 (−0.03 to 0.00) | 0.061 |
| Salt check sheet score | −0.20 (−0.42 to 0.03) | −0.46 (−0.69 to −0.23)‡ | 0.159 |

Mean differences adjusted for age; body mass index; smoking status; alcohol drinking; exercise; hemoglobin A1c; medication for hypertension, diabetes, and dyslipidemia; history of stroke, heart disease, chronic kidney disease, and study field type. Further adjusted for the timing of the urinary test for the analysis of urinary indices.

P values: *<0.05, †<0.01, and ‡<0.001. BP, blood pressure; HDL, high-density lipoprotein; K, potassium; LDL, low-density lipoprotein; Na/K ratio, sodium-to-potassium ratio.

**Supplementary Table 4**. Mean differences (95% confidence intervals) in changes in primary and secondary endpoint parameters between the intensive and standard support groups by age group.

|  | Age (years) | | |  |
| --- | --- | --- | --- | --- |
|  | 18 to 39 | 40 to 64 | ≥65 | P for heterogeneity |
| No. of participants in intensive/standard support groups | 753/1,242 | 1,337/1,150 | 1,974/1,193 |  |
| Primary endpoints |  |  |  |  |
| Urinary Na/K ratio | −0.08 (−0.35 to 0.18) | −0.02 (−0.23 to 0.20) | −0.33 (−0.54 to −0.11)† | 0.066 |
| Estimated daily salt intake, g/day | 0.10 (−0.13 to 0.33) | 0.15 (−0.03 to 0.33) | −0.18 (−0.35 to −0.00)* | 0.004 |
| Estimated daily K intake, mg/day | 35 (−9 to 79) | 27 (−5 to 60) | 40 (9 to 70)* | 0.895 |
| Secondary endpoints |  |  |  |  |
| Systolic BP, mmHg | 0.02 (−0.89 to 0.92) | 1.23 (0.21 to 2.24)* | −1.54 (−2.59 to −0.48)† | 0.005 |
| Diastolic BP, mmHg | 0.29 (−0.45 to 1.03) | 0.99 (0.28 to 1.71)† | 0.28 (−0.38 to 0.93) | 0.898 |
| Body weight, kg | 0.32 (0.04 to 0.59)* | −0.10 (−0.29 to 0.08) | −0.19 (−0.32 to −0.06)† | 0.004 |
| LDL cholesterol, mg/dL | 0.66 (−0.98 to 2.30) | 0.18 (−1.54 to 1.89) | 1.43 (−0.02 to 2.87) | 0.295 |
| HDL cholesterol, mg/dL | 0.35 (−0.34 to 1.04) | 0.72 (0.11 to 1.32)* | 0.23 (−0.32 to 0.78) | 0.935 |
| Triglycerides, mg/dL | 1.41 (−4.57 to 7.39) | 2.40 (−4.82 to 9.62) | −5.49 (−9.96 to −1.01)* | 0.112 |
| Blood glucose, mg/dL | −2.90 (−4.22 to −1.57)‡ | −2.07 (−3.13 to −1.02)‡ | 0.17 (−0.68 to 1.01) | 0.002 |
| Hemoglobin A1c, % | −0.05 (−0.06 to −0.03)‡ | −0.04 (−0.07 to −0.02)† | −0.01 (−0.03 to 0.01) | 0.233 |
| Salt check sheet score | −0.07 (−0.42 to 0.27) | −0.12 (−0.39 to 0.15) | −0.55 (−0.80 to −0.30)‡ | 0.061 |

Mean differences adjusted for age; sex; body mass index; smoking status; alcohol drinking; exercise; hemoglobin A1c; medication for hypertension, diabetes, and dyslipidemia; history of stroke, heart disease, chronic kidney disease, and study field type. Further adjusted for the timing of the urinary test for the analysis of urinary indices.

P values: *<0.05, †<0.01, and ‡<0.001. BP, blood pressure; HDL, high-density lipoprotein; K, potassium; LDL, low-density lipoprotein; Na/K ratio, sodium-to-potassium ratio.

**Supplementary Table 5**. Mean differences (95% confidence intervals) in changes in primary and secondary endpoint parameters between the intensive and standard support groups by quartiles of the baseline urinary Na/K ratio.

|  | Quartiles of urinary Na/K ratio | | | |  |
| --- | --- | --- | --- | --- | --- |
|  | Q1 (−2.06) | Q2 (2.07 to 3.08) | Q3 (3.09 to 4.60) | Q4 (4.61−) | P for heterogeneity |
| No. of participants in intensive/standard support groups | 1,003/911 | 1,037/875 | 977/935 | 1,047/864 |  |
| Primary endpoints |  |  |  |  |  |
| Urinary Na/K ratio | −0.12 (−0.28 to 0.05) | −0.23 (−0.43 to −0.04)* | 0.16 (−0.03 to 0.35) | −0.09 (−0.41 to 0.23) | 0.622 |
| Estimated daily salt intake, g/day | 0.00 (−0.21 to 0.21) | 0.00 (−0.19 to 0.19) | 0.19 (−0.01 to 0.38) | 0.03 (−0.18 to 0.24) | 0.721 |
| Estimated daily K intake, mg/day | 37 (−6 to 81) | 41 (1 to 80)* | 7 (−29 to 44) | 18 (−18 to 54) | 0.279 |
| Secondary endpoints |  |  |  |  |  |
| Systolic BP, mmHg | 0.77 (−0.40 to 1.94) | −0.67 (−1.84 to 0.51) | −0.11 (−1.28 to 1.07) | −1.16 (−2.40 to 0.08) | 0.005 |
| Diastolic BP, mmHg | 0.72 (−0.09 to 1.53) | 0.32 (−0.50 to 1.14) | 0.43 (−0.38 to 1.24) | 0.04 (−0.77 to 0.86) | 0.100 |
| Body weight, kg | −0.11 (−0.33 to 0.12) | 0.04 (−0.17 to 0.25) | 0.06 (−0.15 to 0.27) | −0.27 (−0.49 to −0.06)* | 0.349 |
| LDL cholesterol, mg/dL | 0.38 (−1.53 to 2.29) | 0.91 (−0.91 to 2.73) | 0.33 (−1.49 to 2.15) | 1.66 (−0.16 to 3.48) | 0.479 |
| HDL cholesterol, mg/dL | 0.49 (−0.22 to 1.20) | 0.36 (−0.32 to 1.04) | 0.28 (−0.41 to 0.97) | 0.48 (−0.24 to 1.19) | 0.445 |
| Triglycerides, mg/dL | −1.55 (−7.02 to 3.91) | −1.37 (−7.34 to 4.60) | −2.52 (−10.78 to 5.74) | −1.75 (−8.61 to 5.10) | 0.964 |
| Blood glucose, mg/dL | −2.16 (−3.43 to −0.88)† | −1.15 (−2.45 to 0.16) | −0.87 (−1.97 to 0.22) | 0.12 (−1.05 to 1.29) | 0.024 |
| Hemoglobin A1c, % | −0.06 (−0.09 to −0.03)‡ | −0.02 (−0.04 to 0.01) | −0.02 (−0.05 to 0.00)* | −0.01 (−0.04 to 0.01) | 0.209 |
| Salt check sheet score | −0.03 (−0.37 to 0.31) | −0.22 (−0.55 to 0.11) | −0.56 (−0.87 to −0.25)‡ | −0.40 (−0.71 to −0.09)* | 0.017 |

Mean differences adjusted for age; sex; body mass index; smoking status; alcohol drinking; exercise; hemoglobin A1c; medication for hypertension, diabetes, and dyslipidemia; history of stroke, heart disease, chronic kidney disease, and study field type. Further adjusted for the timing of the urinary test for the analysis of urinary indices.

P values: *<0.05, †<0.01, and ‡<0.001. BP, blood pressure; HDL, high-density lipoprotein; K, potassium; LDL, low-density lipoprotein; Na/K ratio, sodium-to-potassium ratio.

**Supplementary Table 6**. Mean differences (95% confidence intervals) in changes in primary and secondary endpoint parameters between the intensive and standard support groups by quartiles of baseline estimated salt intake.

|  | Quartiles of estimated salt intake (g/day) | | | |  |
| --- | --- | --- | --- | --- | --- |
|  | Q1 (−7.47) | Q2 (7.48−8.77) | Q3 (8.78−10.14) | Q4 (10.15−) | P for heterogeneity |
| No. of participants in intensive/standard support groups | 1,014/899 | 1,063/849 | 1,006/906 | 981/931 |  |
| Primary endpoints |  |  |  |  |  |
| Urinary Na/K ratio | −0.04 (−0.24 to 0.17) | −0.10 (−0.31 to 0.11) | −0.33 (−0.57 to −0.09)† | −0.20 (−0.50 to 0.11) | 0.225 |
| Estimated daily salt intake, g/day | −0.08 (−0.26 to 0.10) | 0.02 (−0.15 to 0.18) | −0.13 (−0.30 to 0.03) | 0.08 (−0.14 to 0.30) | 0.280 |
| Estimated daily K intake, mg/day | 11 (−24 to 46) | 18 (−19 to 56) | 34 (−4 to 71) | 43 (0 to 86) | 0.054 |
| Secondary endpoints |  |  |  |  |  |
| Systolic BP, mmHg | 0.67 (−0.49 to 1.83) | 1.07 (−0.12 to 2.26) | −0.46 (−1.64 to 0.72) | −1.98 (−3.20 to −0.76)† | <0.001 |
| Diastolic BP, mmHg | 0.62 (−0.17 to 1.40) | 1.21 (0.41 to 2.01)† | 0.48 (−0.35 to 1.32) | −0.49 (−1.31 to 0.34) | 0.009 |
| Body weight, kg | −0.14 (−0.34 to 0.07) | 0.18 (−0.03 to 0.39) | −0.03 (−0.24 to 0.18) | −0.26 (−0.49 to −0.02)* | 0.195 |
| LDL cholesterol, mg/dL | 0.42 (−1.40 to 2.23) | 1.05 (−0.79 to 2.90) | 0.58 (−1.24 to 2.39) | 0.92 (−0.94 to 2.78) | 0.991 |
| HDL cholesterol, mg/dL | 0.62 (−0.12 to 1.35) | 0.52 (−0.17 to 1.21) | 0.37 (−0.29 to 1.03) | 0.21 (−0.49 to 0.91) | 0.732 |
| Triglycerides, mg/dL | −2.11 (−7.46 to 3.23) | 2.07 (−3.31 to 7.46) | −1.42 (−8.26 to 5.41) | −5.82 (−14.59 to 2.95) | 0.311 |
| Blood glucose, mg/dL | −2.15 (−3.33 to −0.96)‡ | 0.14 (−1.05 to 1.33) | −1.62 (−2.89 to −0.36)* | −0.43 (−1.64 to 0.78) | 0.112 |
| Hemoglobin A1c, % | −0.06 (−0.08 to −0.03)‡ | −0.01 (−0.03 to 0.01) | −0.03 (−0.06 to −0.01)* | −0.02 (−0.05 to 0.01) | 0.099 |
| Salt check sheet score | −0.12 (−0.44 to 0.21) | −0.35 (−0.66 to −0.04)* | −0.34 (−0.66 to −0.02)* | −0.41 (−0.74 to −0.07)* | 0.224 |

Mean differences adjusted for age; sex; body mass index; smoking status; alcohol drinking; exercise; hemoglobin A1c; medication for hypertension, diabetes, and dyslipidemia; history of stroke, heart disease, chronic kidney disease, and study field type. Further adjusted for the timing of the urinary test for the analysis of urinary indices.

P values: *<0.05, †<0.01, and ‡<0.001. BP, blood pressure; HDL, high-density lipoprotein; K, potassium; LDL, low-density lipoprotein; Na/K ratio, sodium-to-potassium ratio.

**Supplementary Table 7**. Mean differences (95% confidence intervals) in changes in primary and secondary endpoint parameters between the intensive and standard support groups by quartiles of baseline estimated K intake.

|  | Quartiles of estimated K intake (mg/day) | | | |  |
| --- | --- | --- | --- | --- | --- |
|  | Q1 (−1669.31) | Q2 (1669.32−1947.21) | Q3 (1947.22−2279.63) | Q4 (2279.64−) | P for heterogeneity |
| No. of participants in intensive/standard support groups | 1,050/863 | 1,023/889 | 1,001/911 | 990/922 |  |
| Primary endpoints |  |  |  |  |  |
| Urinary Na/K ratio | −0.01 (−0.34 to 0.32) | 0.04 (−0.20 to 0.27) | −0.07 (−0.29 to 0.15) | −0.25 (−0.43 to −0.07)† | 0.106 |
| Estimated daily salt intake, g/day | 0.00 (−0.19 to 0.19) | 0.03 (−0.17 to 0.22) | −0.11 (−0.33 to 0.11) | 0.03 (−0.21 to 0.26) | 0.986 |
| Estimated daily K intake, mg/day | −18 (−48 to 12) | 8 (−23 to 39) | −7 (−42 to 29) | 69 (27 to 111)† | <0.001 |
| Secondary endpoints |  |  |  |  |  |
| Systolic BP, mmHg | 0.53 (−0.65 to 1.71) | −0.66 (−1.84 to 0.53) | −0.72 (−1.99 to 0.54) | −0.05 (−1.21 to 1.12) | 0.636 |
| Diastolic BP, mmHg | 1.30 (0.52 to 2.08)† | −0.12 (−0.91 to 0.68) | 0.03 (−0.83 to 0.89) | 0.40 (−0.43 to 1.22) | 0.258 |
| Body weight, kg | −0.26 (−0.46 to −0.06)* | 0.11 (−0.10 to 0.31) | 0.03 (−0.20 to 0.26) | −0.09 (−0.32 to 0.14) | 0.468 |
| LDL cholesterol, mg/dL | −0.05 (−1.89 to 1.80) | 1.66 (−0.06 to 3.37) | 1.65 (−0.37 to 3.66) | 0.07 (−1.75 to 1.88) | 0.865 |
| HDL cholesterol, mg/dL | 0.46 (−0.30 to 1.21) | 0.17 (−0.51 to 0.84) | 0.52 (−0.16 to 1.21) | 0.42 (−0.26 to 1.10) | 0.284 |
| Triglycerides, mg/dL | 2.86 (−3.12 to 8.84) | −1.68 (−7.34 to 3.97) | −9.43 (−16.45 to −2.41)† | 1.35 (−6.82 to 9.52) | 0.342 |
| Blood glucose, mg/dL | 0.04 (−1.16 to 1.24) | −1.17 (−2.27 to −0.06)* | −1.48 (−2.67 to −0.29)* | −1.83 (−3.18 to −0.48)† | 0.050 |
| Hemoglobin A1c, % | −0.01 (−0.04 to 0.01) | −0.03 (−0.05 to −0.01)* | −0.03 (−0.06 to 0.00)* | −0.03 (−0.06 to 0.00)* | 0.980 |
| Salt check sheet score | −0.33 (−0.63 to −0.03)* | −0.25 (−0.56 to 0.06) | −0.47 (−0.81 to −0.12)† | −0.23 (−0.56 to 0.11) | 0.895 |

Mean differences adjusted for age; sex; body mass index; smoking status; alcohol drinking; exercise; hemoglobin A1c; medication for hypertension, diabetes, and dyslipidemia; history of stroke, heart disease, chronic kidney disease, and study field type. Further adjusted for the timing of the urinary test for the analysis of urinary indices.

P values: *<0.05, †<0.01, and ‡<0.001. BP, blood pressure; HDL, high-density lipoprotein; K, potassium; LDL, low-density lipoprotein; Na/K ratio, sodium-to-potassium ratio.

**Supplementary Table 8**. Mean differences (95% confidence intervals) in changes in primary and secondary endpoint parameters between the intensive and standard support groups by baseline alcohol drinking.

|  | Alcohol drinking | | | |  |
| --- | --- | --- | --- | --- | --- |
|  | Nondrinker | Light drinker | Moderate drinker | Heavy drinker | P for heterogeneity |
| No. of participants in intensive/standard support groups | 1,913/1,762 | 1,063/816 | 756/694 | 332/313 |  |
| Primary endpoints |  |  |  |  |  |
| Urinary Na/K ratio | −0.05 (−0.24 to 0.13) | −0.18 (−0.43 to 0.06) | −0.25 (−0.55 to 0.05) | −0.29 (−0.73 to 0.16) | 0.425 |
| Estimated daily salt intake, g/day | 0.08 (−0.07 to 0.24) | −0.10 (−0.31 to 0.11) | −0.07 (−0.31 to 0.18) | 0.21 (−0.18 to 0.59) | 0.775 |
| Estimated daily K intake, mg/day | 36 (8 to 64)* | 13 (−25 to 52) | 36 (−11 to 83) | 70 (−1 to 141) | 0.262 |
| Secondary endpoints |  |  |  |  |  |
| Systolic BP, mmHg | 0.31 (−0.56 to 1.18) | −1.02 (−2.23 to 0.18) | −0.17 (−1.50 to 1.16) | −0.46 (−2.43 to 1.52) | 0.317 |
| Diastolic BP, mmHg | 0.66 (0.07 to 1.24)* | 0.07 (−0.72 to 0.87) | 0.69 (−0.27 to 1.66) | 0.12 (−1.26 to 1.50) | 0.967 |
| Body weight, kg | −0.06 (−0.21 to 0.10) | −0.06 (−0.26 to 0.14) | −0.14 (−0.41 to 0.12) | 0.07 (−0.31 to 0.45) | 0.998 |
| LDL cholesterol, mg/dL | 0.85 (−0.52 to 2.21) | 1.20 (−0.57 to 2.96) | 0.83 (−1.16 to 2.82) | −1.01 (−4.49 to 2.48) | 0.565 |
| HDL cholesterol, mg/dL | 0.29 (−0.19 to 0.76) | 0.75 (0.07 to 1.42)* | 0.70 (−0.13 to 1.53) | −0.21 (−1.78 to 1.36) | 0.874 |
| Triglycerides, mg/dL | 1.07 (−3.19 to 5.32) | −4.89 (−10.62 to 0.85) | −5.49 (−13.42 to 2.44) | 1.87 (−18.16 to 21.89) | 0.800 |
| Blood glucose, mg/dL | −0.34 (−1.21 to 0.52) | −1.32 (−2.32 to −0.33)† | −2.77 (−4.35 to −1.18)† | 0.78 (−1.73 to 3.29) | 0.163 |
| Hemoglobin A1c, % | −0.02 (−0.04 to 0.00)* | −0.02 (−0.05 to 0.00) | −0.04 (−0.07 to −0.01)† | −0.03 (−0.07 to 0.02) | 0.621 |
| Salt check sheet score | −0.26 (−0.49 to −0.02)* | −0.24 (−0.57 to 0.09) | −0.44 (−0.80 to −0.08)* | −0.39 (−0.96 to 0.19) | 0.650 |

Mean differences adjusted for age; sex; body mass index; smoking status; exercise; hemoglobin A1c; medication for hypertension, diabetes, and dyslipidemia; history of stroke, heart disease, chronic kidney disease, and study field type. Further adjusted for the timing of the urinary test for the analysis of urinary indices. P values: *<0.05, †<0.01, and ‡<0.001. BP, blood pressure; HDL, high-density lipoprotein; K, potassium; LDL, low-density lipoprotein; Na/K ratio, sodium-to-potassium ratio. Alcohol drinking categories were evaluated using questionnaires on the frequency and quantity of alcohol consumption, categorizing individuals as non-drinkers (almost never), light drinkers (daily or occasionally, <180 ml), moderate drinkers (daily or occasionally, 180–360 ml), and heavy drinkers (daily or occasionally, >360 ml).

**Supplementary Table 9**. Mean differences (95% confidence intervals) in changes in primary and secondary endpoint parameters between the intensive and standard support groups by quartiles of baseline body mass index category.

|  | Body mass index (kg/m^2^) | | |  |
| --- | --- | --- | --- | --- |
|  | <18.5 | 18.5 to 24.9 | ≥25.0 | P for heterogeneity |
| No. of participants in intensive/standard support groups | 311/283 | 2,724/2,471 | 1,029/831 |  |
| Primary endpoints |  |  |  |  |
| Urinary Na/K ratio | −0.37 (−0.84 to 0.11) | −0.21 (−0.36 to −0.05)† | 0.13 (−0.14 to 0.40) | 0.016 |
| Estimated daily salt intake, g/day | 0.02 (−0.33 to 0.36) | −0.07 (−0.20 to 0.05) | 0.28 (0.04 to 0.52)* | 0.059 |
| Estimated daily K intake, mg/day | 42 (−25 to 110) | 33 (9 to 56)† | 19 (−22 to 59) | 0.306 |
| Secondary endpoints |  |  |  |  |
| Systolic BP, mmHg | 0.74 (−1.30 to 2.78) | 0.05 (−0.67 to 0.76) | −1.06 (−2.32 to 0.20) | 0.038 |
| Diastolic BP, mmHg | −0.38 (−1.76 to 1.00) | 0.70 (0.22 to 1.18)† | −0.02 (−0.89 to 0.85) | 0.527 |
| Body weight, kg | −0.12 (−0.38 to 0.14) | −0.05 (−0.16 to 0.07) | −0.11 (−0.39 to 0.18) | 0.368 |
| LDL cholesterol, mg/dL | 0.48 (−2.54 to 3.50) | 0.60 (−0.48 to 1.68) | 1.10 (−0.95 to 3.15) | 0.521 |
| HDL cholesterol, mg/dL | 0.90 (−0.59 to 2.40) | 0.50 (0.08 to 0.93)* | 0.08 (−0.55 to 0.71) | 0.157 |
| Triglycerides, mg/dL | −9.54 (−17.16 to −1.93)* | −2.89 (−6.47 to 0.70) | 4.07 (−5.08 to 13.21) | 0.013 |
| Blood glucose, mg/dL | −1.41 (−3.59 to 0.77) | −0.74 (−1.33 to −0.16)* | −1.66 (−3.35 to 0.03) | 0.216 |
| Hemoglobin A1c, % | 0.00 (−0.04 to 0.03) | −0.02 (−0.03 to −0.01)† | −0.05 (−0.09 to −0.02)† | 0.012 |
| Salt check sheet score | −0.25 (−0.79 to 0.29) | −0.30 (−0.49 to −0.12)† | −0.35 (−0.72 to 0.01) | 0.590 |

Mean differences adjusted for age; sex; body mass index; smoking status; alcohol drinking; exercise; hemoglobin A1c; medication for hypertension, diabetes, and dyslipidemia; history of stroke, heart disease, chronic kidney disease, and study field type. Further adjusted for the timing of the urinary test for the analysis of urinary indices.

P values: *<0.05, †<0.01, and ‡<0.001. BP, blood pressure; HDL, high-density lipoprotein; K, potassium; LDL, low-density lipoprotein; Na/K ratio, sodium-to-potassium ratio.

**Supplementary Table 10**. Mean differences (95% confidence intervals) in changes in primary and secondary endpoint parameters between the intensive and standard support groups by baseline hypertension status.

|  | Hypertension | |  |
| --- | --- | --- | --- |
|  | Absence | Presence | P for heterogeneity |
| No. of participants in intensive/standard support groups | 2,552/2,544 | 1,512/1,041 |  |
| Primary endpoints |  |  |  |
| Urinary Na/K ratio | −0.14 (−0.29 to 0.01) | −0.16 (−0.41 to 0.08) | 0.999 |
| Estimated daily salt intake, g/day | 0.03 (−0.10 to 0.15) | −0.01 (−0.22 to 0.20) | 0.742 |
| Estimated daily K intake, mg/day | 28 (4 to 52)* | 39 (5 to 73)* | 0.731 |
| Secondary endpoints |  |  |  |
| Systolic BP, mmHg | 0.44 (−0.18 to 1.06) | −1.59 (−2.83 to −0.35)* | <0.001 |
| Diastolic BP, mmHg | 0.50 (0.04 to 0.96)* | 0.26 (−0.53 to 1.04) | 0.744 |
| Body weight, kg | 0.00 (−0.14 to 0.13) | −0.18 (−0.36 to 0.00) | 0.079 |
| LDL cholesterol, mg/dL | 0.40 (−0.68 to 1.48) | 1.34 (−0.36 to 3.04) | 0.297 |
| HDL cholesterol, mg/dL | 0.50 (0.08 to 0.92)* | 0.29 (−0.32 to 0.90) | 0.694 |
| Triglycerides, mg/dL | 0.46 (−3.50 to 4.41) | −5.87 (−12.12 to 0.38) | 0.144 |
| Blood glucose, mg/dL | −0.86 (−1.56 to −0.16)* | −1.21 (−2.31 to −0.10)* | 0.635 |
| Hemoglobin A1c, % | −0.03 (−0.04 to −0.01)‡ | −0.02 (−0.05 to 0.00) | 0.892 |
| Salt check sheet score | −0.24 (−0.43 to −0.04)* | −0.42 (−0.71 to −0.14)† | 0.295 |

Mean differences adjusted for age; sex; body mass index; smoking status; alcohol drinking; exercise; hemoglobin A1c; medication for diabetes and dyslipidemia; history of stroke, heart disease, chronic kidney disease, and study field type. Further adjusted for the timing of the urinary test for the analysis of urinary indices. P values: *<0.05, †<0.01, and ‡<0.001. BP, blood pressure; HDL, high-density lipoprotein; K, potassium; LDL, low-density lipoprotein; Na/K ratio, sodium-to-potassium ratio. Hypertension was defined as systolic BP ≥140 mmHg, diastolic BP ≥90 mmHg, or antihypertensive medication use.

**Supplementary Table 11**. Mean differences (95% confidence intervals) in changes in primary and secondary endpoint parameters between the intensive and standard support groups by baseline diabetes mellitus status.

|  | Diabetes mellitus | |  |
| --- | --- | --- | --- |
|  | Absence | Presence | P for heterogeneity |
| No. of participants in intensive/standard support groups | 3,684/3,338 | 380/247 |  |
| Primary endpoints |  |  |  |
| Urinary Na/K ratio | −0.13 (−0.26 to 0.00) | −0.31 (−0.79 to 0.16) | 0.784 |
| Estimated daily salt intake, g/day | 0.01 (−0.10 to 0.12) | 0.04 (−0.37 to 0.46) | 0.473 |
| Estimated daily K intake, mg/day | 28 (8 to 49)† | 68 (−6 to 142) | 0.500 |
| Secondary endpoints |  |  |  |
| Systolic BP, mmHg | −0.07 (−0.67 to 0.54) | −1.63 (−4.10 to 0.85) | 0.233 |
| Diastolic BP, mmHg | 0.54 (0.13 to 0.96)* | −0.83 (−2.35 to 0.70) | 0.152 |
| Body weight, kg | −0.04 (−0.15 to 0.07) | −0.31 (−0.71 to 0.09) | 0.343 |
| LDL cholesterol, mg/dL | 0.70 (−0.24 to 1.65) | 1.36 (−2.26 to 4.97) | 0.434 |
| HDL cholesterol, mg/dL | 0.45 (0.09 to 0.82)* | 0.20 (−1.00 to 1.40) | 0.692 |
| Triglycerides, mg/dL | −2.78 (−5.94 to 0.38) | 10.94 (−9.65 to 31.53) | 0.020 |
| Blood glucose, mg/dL | −0.96 (−1.37 to −0.56)‡ | −0.77 (−5.91 to 4.38) | 0.378 |
| Hemoglobin A1c, % | −0.03 (−0.04 to −0.02)‡ | −0.02 (−0.14 to 0.10) | 0.364 |
| Salt check sheet score | −0.26 (−0.42 to −0.09)† | −0.87 (−1.49 to −0.24)† | 0.066 |

Mean differences adjusted for age; sex; body mass index; smoking status; alcohol drinking; exercise; hemoglobin A1c; medication for hypertension and dyslipidemia; history of stroke, heart disease, chronic kidney disease, and study field type. Further adjusted for the timing of the urinary test for the analysis of urinary indices. P values: *<0.05, †<0.01, and ‡<0.001. BP, blood pressure; HDL, high-density lipoprotein; K, potassium; LDL, low-density lipoprotein; Na/K ratio, sodium-to-potassium ratio. Diabetes mellitus was defined as fasting blood glucose ≥126 mg/dl, hemoglobin A1c ≥6.5%, or antidiabetic medication use.

**Supplementary Table 12**. Mean differences (95% confidence intervals) in changes in primary and secondary endpoint parameters between the intensive and standard support groups by study field type.

|  | Study field | |  |
| --- | --- | --- | --- |
|  | Municipalities | Workplaces | P for heterogeneity |
| No. of participants in intensive/standard support groups | 2,903/1,685 | 1,161/1,900 |  |
| Primary endpoints |  |  |  |
| Urinary Na/K ratio | −0.24 (−0.42 to −0.05)* | −0.03 (−0.22 to 0.16) | 0.140 |
| Estimated daily salt intake, g/day | −0.12 (−0.26 to 0.03) | 0.16 (−0.02 to 0.34) | 0.015 |
| Estimated daily K intake, mg/day | 32 (6 to 58)* | 31 (−3 to 64) | 0.895 |
| Secondary endpoints |  |  |  |
| Systolic BP, mmHg | −0.78 (−1.64 to 0.09) | 0.54 (−0.21 to 1.29) | 0.022 |
| Diastolic BP, mmHg | 0.20 (−0.34 to 0.75) | 1.03 (0.42 to 1.63)† | 0.096 |
| Body weight, kg | −0.25 (−0.37 to −0.13)‡ | 0.22 (0.02 to 0.42)* | <0.001 |
| LDL cholesterol, mg/dL | 1.14 (−0.10 to 2.38) | 0.53 (−0.84 to 1.90) | 0.369 |
| HDL cholesterol, mg/dL | 0.36 (−0.10 to 0.83) | 0.50 (−0.03 to 1.03) | 0.586 |
| Triglycerides, mg/dL | −0.74 (−5.02 to 3.54) | −1.22 (−6.74 to 4.29) | 0.542 |
| Blood glucose, mg/dL | −0.09 (−0.82 to 0.64) | −3.14 (−4.26 to −2.02)‡ | <0.001 |
| Hemoglobin A1c, % | −0.02 (−0.04 to −0.01)* | −0.03 (−0.05 to −0.01)‡ | 0.628 |
| Salt check sheet score | −0.40 (−0.61 to −0.20)‡ | −0.13 (−0.39 to 0.13) | 0.130 |

Mean differences adjusted for age; sex; body mass index; smoking status; alcohol drinking; exercise; hemoglobin A1c; medication for hypertension, diabetes, and dyslipidemia; and history of stroke, heart disease, and chronic kidney disease. Further adjusted for the timing of the urinary test for the analysis of urinary indices.

P values: *<0.05, †<0.01, and ‡<0.001. BP, blood pressure; HDL, high-density lipoprotein; K, potassium; LDL, low-density lipoprotein; Na/K ratio, sodium-to-potassium ratio.

**Supplementary Table 13**. Mean differences (95% confidence intervals) in changes in primary endpoint parameters between the intensive and standard support groups by the timing of the urinary test

|  | Timing of the urinary test | |  |
| --- | --- | --- | --- |
|  | Morning at home | Daytime during health check-up | P for heterogeneity |
| No. of participants in intensive/standard support groups | 2,121/1,739 | 1,943/1,846 |  |
| Primary endpoints |  |  |  |
| Urinary Na/K ratio | -0.23 (-0.43 to -0.03)* | -0.05 (-0.22 to 0.12) | 0.212 |
| Estimated daily salt intake, g/day | -0.10 (-0.24 to 0.04) | 0.15 (-0.03 to 0.32) | 0.033 |
| Estimated daily K intake, mg/day | 30 (4 to 57)* | 32 (-1 to 65) | 0.911 |

Mean differences adjusted for age; body mass index; smoking status; alcohol drinking; exercise; hemoglobin A1c; medication for hypertension, diabetes, and dyslipidemia; history of stroke, heart disease, chronic kidney disease, and study field type.

P values: *<0.05, †<0.01, and ‡<0.001. K, potassium; Na/K ratio, sodium-to-potassium ratio.

**Appendix**. Members of the PoSPIP Research Group

**Chairpersons**: Katsuyuki Miura (Shiga University of Medical Science, Otsu, Japan) and Koichi Node (Saga University, Saga, Japan)

**Committee Members**: Koichi Node (Saga University, Saga, Japan), Hiromi Rakugi (Osaka Rosai Hospital, Sakai, Japan), Hiroshi Itoh (Keio University, Tokyo, Japan), Katsuyuki Miura (Shiga University of Medical Science, Otsu, Japan), and Tomonori Okamura (Keio University School of Medicine, Tokyo, Japan)

**Research Members**:

**Field working group:** Takuya Tsuchihashi (Steel Memorial Yawata Hospital, Kitakyushu, Japan), Mitsuru Ohishi, Yuichi Akasaki (Kagoshima University, Kagoshima, Japan), Naoyuki Hasebe, Naoki Nakagawa (Asahikawa University, Asahikawa, Japan), Miho Kusaka (Kusaka Clinic, Kure, Japan), Masaru Sakurai (Kanagawa Medical University, Kanagawa, Japan), Masakazu Nakamura, Teruko Kawabata (Health Promotion Research Center, Tokyo, Japan), Nobuo Yoshiike, Tatsuya Koyama (Aomori University of Health and Welfare, Aomori, Japan), Kensuke Noma (Noma Clinic, Kure, Japan)

**Health guidance group:** Katushi Yoshita (Osaka Metropolitan University, Osaka, Japan), Takuya Kishi (International University of Health and Welfare, Okawa, Japan), Rumi Tsukinogi (Tokyo Medical and Dental University, Tokyo, Japan), Takashi Hisamatsu (Okayama University, Okayama, Japan), Keiko Kondo (Shiga University of Medical Science, Otsu, Japan)

**Food environment improvement group:** Yukari Takemi, Akiko Kubo (Kagawa Nutrition University, Sakado, Japan), Hitomi Hayabuchi, Masanori Ota (Fukuoka Women’s University, Fukuoka, Japan), Ikuko Sakata (Seinan Jo Gakuin University, Kitakyushu, Japan), Keiko Sakaguchi (Shukutoku University, Chiba, Japan)

**Data management and analysis group**: Takayoshi Ohkubo (Teikyo University School of Medicine, Tokyo, Japan), Yuichiro Yano (Juntendo University, Tokyo, Japan), Takashi Hisamatsu (Okayama University, Okayama, Japan), Aya Kadota (Shiga University of Medical Science, Otsu, Japan)

**Medical economic analysis group**: Rei Goto (Keio University Business School, Tokyo, Japan), Shohei Okamoto (Tokyo Metropolitan Institute for Geriatrics and Gerontology, Tokyo, Japan)

**Technology evaluation group**: Atsuhi Hozawa, Mana Kogure (Tohoku University, Sendai, Japan), Yasuharu Tabara (Shizuoka Graduate University of Public Health)

**Administrative office**: Kaori Kitaoka, Yukiko Okami (Shiga University of Medical Science, Otsu, Japan)
